# Mapping psychological heterogeneity in military cadets: a multi-method framework combining network analysis, latent profiles, and risk nomograms

**DOI:** 10.3389/fpsyt.2026.1920552

**Published:** 2026-08-27

**Authors:** Charis Ntakolia, Vasiliki Yotsidi

**Affiliations:** 1 Department of Aeronautical Studies, Sector of Materials Engineering, Machining Technology and Production Management, Hellenic Air Force Academy, Dekeleia Base, Acharnes, Attica, Greece; 2 Department of Psychology, Panteion University of Social and Political Sciences; Kallithea, Attica, Greece

**Keywords:** distress, latent profile analysis, mental health, military training, network analysis, organizational skills, personality traits, resilience

## Abstract

**Introduction:**

Military training imposes sustained psychological demands, yet cadet cohorts are typically treated as homogeneous, which obscures individual differences in distress, resilience, and adaptation. This study examined psychological adaptation and its heterogeneity across an initial training period.

**Methods:**

Ninety-two cadets completed the DASS-21, the CD-RISC-25, and personality measures on three occasions spanning five months. A multi-method framework combined four complementary analyses: longitudinal measurement invariance testing through confirmatory factor analysis and Tucker's congruence coefficients, psychological network estimation with graphical LASSO regularisation, latent profile analysis, and logistic regression nomograms developed as clinical screening tools. A pre-specified sensitivity analysis replaced the baseline dynamic indicators with T2 minus T0 change scores while keeping trait level inputs unchanged.

**Results:**

The CD-RISC-25 demonstrated excellent factorial stability (mean congruence = .971), whereas the DASS-21 showed fair stability (mean congruence = .867). Depression and stress formed the strongest partial correlation edge (r = .54, bootstrap inclusion = 100%), with resilience acting as a bridging node between distress and personality domains. Latent profile analysis identified five cadet profiles with high classification entropy (.973) and significant differentiation across 17 outcome variables. The change score solution again yielded five profiles (entropy = .955) but showed only moderate concordance with the primary solution (adjusted Rand index = .245). The academic performance nomogram achieved the highest cross-validated accuracy (AUC = .682).

**Discussion:**

Baseline and trajectory typologies capture complementary, non-redundant facets of cadet heterogeneity, supporting an integrated account of adaptation to military training and offering practical tools for early identification of at risk cadets, although predictive accuracy remains modest.

## 1 Introduction

Military academy training represents one of the most demanding educational and occupational settings encountered in early adulthood. Cadets are required to meet academic standards, sustain intensive physical conditioning, and master a hierarchical professional culture while simultaneously coping with sleep restriction, separation from family, restricted autonomy, and pervasive evaluation pressure (1, 2). Empirical work has repeatedly shown that exposure to this constellation of demands is associated with elevated symptoms of depression, anxiety, and stress, particularly during the basic training phase (3, 4), and that the resulting strain contributes to attrition, overuse injury, and impaired operational readiness (5, 6). Understanding how cadets adapt psychologically to this environment is therefore not only a question of student welfare but also a question of force preparedness.

Three classes of psychological variables have emerged as central to cadet adjustment in this literature: personality traits, psychological distress, and resilience. The Big Five personality dimensions, particularly conscientiousness and emotional stability, have been consistently linked to academic and occupational performance across diverse settings (7, 8), and within military samples specifically have been associated with cadet adaptation, leadership emergence, and resistance to attrition (9, 10). Psychological distress, typically operationalised through the depression, anxiety, and stress subscales of the DASS-21 (11, 12), captures the symptomatic burden imposed by training demands and is a robust short-term predictor of dropout, disciplinary infractions, and reduced wellbeing (1, 13). Resilience, conceptualised as the capacity to maintain or recover psychosocial functioning under adversity (14, 15), has received particular attention in military contexts because it is empirically related both to performance under stress and to the trajectory of distress symptoms during prolonged training (16, 17).

Beyond their relevance to wellbeing, these constructs also map onto the broader organisational psychology literature on work performance. Personality and resilience predict not only task performance but also organisational citizenship behaviour (OCB), defined as discretionary, role-extending contributions that support the effective functioning of the organisation (18–20). In military contexts, OCB overlaps with concepts such as group cohesion, voluntary mentoring, and unit-supportive behaviours that are critical for both training success and operational effectiveness (10, 21). Examining personality, distress, resilience, and OCB together within a single cohort therefore allows the joint study of two outcomes that have typically been treated in separate literatures: psychological adaptation (a clinical concern) and discretionary work behaviour (an organisational concern).

Existing research on cadet psychological adaptation has nonetheless been constrained by three recurring limitations. First, most studies have adopted a variable-centred approach, examining how individual predictors relate to individual outcomes through correlation, regression, or structural equation modelling (2, 9). Such designs are well suited to identifying average effects but are not designed to recover qualitatively distinct subgroups of cadets, even though person-centred evidence from related literatures suggests that such heterogeneity is the rule rather than the exception (22). Second, studies typically examine constructs in isolation rather than as a system of mutually conditioning variables, missing the interdependence captured by network-analytic frameworks (23, 24). Third, the translational gap between empirical findings and the clinical or administrative tools available to training officers has rarely been bridged: even when significant predictors are identified, they are seldom packaged into screening instruments that can be used at intake (25, 26). The consequence is a literature that is rich in mean-level associations but comparatively poor in tools that frontline staff can deploy to identify cadets who may benefit from early intervention.

The present study addresses these gaps by integrating four complementary analytical perspectives within a single longitudinal cohort of 92 Hellenic Air Force Academy cadets assessed at three timepoints (T0, T1, T2, spanning the first five months of training). First, longitudinal measurement invariance of the DASS-21 and CD-RISC-25 is tested to ensure that within-person change observed across the training period reflects substantive change rather than measurement artefact (27, 28). Second, two psychological networks are estimated via graphical LASSO regularisation (24, 29): a cross-sectional network mapping the partial correlation structure among personality, distress, resilience, OCB, leadership, and performance outcomes, and a temporal network mapping the evolution of distress and resilience across T0, T1, and T2. Third, latent profile analysis using Gaussian mixture models (22, 30) identifies subgroups of cadets defined by their baseline psychological configuration, and a pre-specified sensitivity analysis substitutes change scores (T2 minus T0) for the dynamic indicators to test whether the typology is timepoint-robust. Fourth, logistic-regression risk nomograms (25, 31) are constructed as simple, deployable screening tools for academic, athletic, and OCB outcomes. By combining these four perspectives, the study aims to deliver an integrated mapping of psychological heterogeneity in military cadets that is at once theoretically grounded, methodologically rigorous, and clinically actionable.

The four analytical steps outlined above should not be regarded as independent contributions but as a single methodological sequence, in which each step addresses a component of the overarching research question — namely, the identification of cadets at psychological risk within this cohort and the specification of the measurement, structural, typological, and translational conditions under which such identification can be reliably achieved.

The first step, longitudinal measurement invariance testing, constitutes a psychometric prerequisite for the analyses that follow. By establishing that the DASS-21 and CD-RISC-25 preserve their factorial structure across the three assessment occasions, this step ensures that within-person variation observed at T1 and T2 can be interpreted as substantive psychological change rather than as an artefact of instrumental drift. In the absence of this evidentiary basis, no longitudinal inference drawn in the subsequent steps could be considered interpretatively secure.

The second step, psychological network analysis, employs the validated instruments to characterise the variable-level structural relationships among personality, distress, resilience, leadership, organisational citizenship, and performance indicators, first cross-sectionally at T0 (Network A) and subsequently across the three measurement waves in a temporal formulation (Network B). This step reveals which constructs occupy central and bridging positions within the cadet system, yet it does so at the population level and treats the sample as internally homogeneous.

The third step, latent profile analysis, complements the variable-centred perspective of the network with a person-centred one. Drawing on the same set of baseline indicators, it identifies subgroups of cadets whose joint psychological configurations differ qualitatively from those of their peers, thereby shifting the analytical focus from relationships between variables to differentiation between individuals. A pre-specified sensitivity analysis, in which change-score inputs replace the T0 dynamic indicators, further examines whether the recovered typology is robust to the choice of measurement timepoint.

The fourth step, the construction of logistic-regression risk nomograms, translates the psychological information characterised in the preceding steps into an operationally deployable screening instrument. By expressing the joint predictive value of the baseline indicators as a point-based scoring rule for academic, athletic, and organisational citizenship outcomes, this step renders the descriptive and structural findings actionable within routine institutional practice.

Taken as a whole, the four steps progress from measurement validity, through structural characterisation and person-centred typology, to clinical translation. Each step addresses a question that its predecessor raises but cannot itself resolve, and it is only through their integration that the manuscript delivers an internally consistent account of psychological heterogeneity in the present cadet cohort.

The study sample comprised 92 Hellenic Air Force Academy cadets assessed at three timepoints: T0 (entry), T1 (post-basic training, approximately one month), and T2 (end of semester, approximately five months). The assessed constructs included the Big Five personality traits [BFI-44, T0 (32)], psychological distress [DASS-21, T0/T1/T2 (11, 33]), resilience [CD-RISC-25, T0/T1/T2 (14, 34)], leadership (T0), organisational citizenship behaviour comprising five subscales [altruism, conscientiousness, sportsmanship, courtesy, civic virtue, at T2 (19)], as well as academic and athletic performance scores (T2). Demographic variables included gender, sibling status, living conditions, parental education, air force family background, living area, exercise frequency, choice of academy, and reason for enrolment. This study is part of a comprehensive investigation of the impact of basic training to Hellenic Air Force Academy cadets (35–37).

## 2 Methodology

### 2.1 Participants and questionnaires

The sample comprised N = 92 cadets. The majority of the participants were 18 years old (n = 73), with 16 participants aged 19, 2 aged 20, and 1 aged 21. The sample was predominantly male (n = 62, 67.4%), with 30 female participants (32.6%). Participants were enrolled in standard military training programs and provided informed consent to participate in the study. The independent variables included 32 base features spanning demographics (gender, siblings, living conditions, parental education, air force family, living area, habits, HAFA choice/reason at T0), personality (BFI-44: openness, conscientiousness, extraversion, agreeableness, neuroticism at T0), psychological distress (DASS-21: depression, anxiety, stress at T0, T1, T2), resilience (CD-RISC-25 at T0, T1, T2), leadership at T0, and organizational behavior (OCB-20: altruism, conscientiousness, sportsmanship, courtesy, and civic virtue at T2).

Personality characteristics were assessed using the Big Five Inventory-44 adapted to the Greek population (38). This questionnaire measures five domains: Extraversion, Conscientiousness, Agreeableness, Neuroticism, and Openness. Internal consistency (Cronbach’s α) in the present sample was α = .705 for the total scale,.726 for Extraversion,.722 for Agreeableness,.775 for Conscientiousness,.820 for Neuroticism, and.730 for Openness (35–37).

A self-explanatory leadership questionnaire consisting of six items was developed by initio by the research team to capture leadership-related traits relevant to the military context. The leadership scale demonstrated acceptable internal consistency (α = .723), indicating satisfactory reliability for research purposes (35–37).

Mental distress was operationalized as the total score on the Depression, Anxiety and Stress Scale - 21 Items adapted to the Greek population (39), which captures symptoms across three domains: depression, anxiety, and stress. Higher scores reflect greater psychological discomfort during the initial phase of military training. Internal consistency was good to excellent across scales and timepoints in the present sample: total α = .945,.878, and.946 at T0, T1, and T2, respectively; Depression α = .879,.778, and.888; Anxiety α = .823,.686, and.857; Stress α = .869,.765, and.855 (35–37).

The Connor–Davidson Resilience Scale (CD-RISC-25) is a widely used 25-item self-report measure of resilience, with higher scores indicating greater resilience. In the present study, the Greek version of the instrument was administered (34). The total scale demonstrated good internal consistency at all timepoints (α = .895,.896, and.874 at T0, T1, and T2, respectively) (35–37).

The Organizational Citizenship Behavior (OCB) questionnaire is a 24-item measure of discretionary, extra-role workplace behaviors that support the effective functioning of the organization, evaluated and translated in Greek (40). The instrument is commonly based on the five-dimensional model of OCB assessing altruism, conscientiousness, sportsmanship, courtesy, and civic virtue (18, 19). In the current study, the total scale showed acceptable reliability at T2 (α = .760, 95% CI [.683,.825]). Subscale alphas at T2 were α = .725 for Altruism,.774 for Conscientiousness,.766 for Sportsmanship,.732 for Civic Virtue, and.646 for Courtesy (35–37).

### 2.2 Step 1: measurement invariance

Longitudinal measurement invariance was assessed for the DASS-21 and CD-RISC-25 to verify that these instruments measured the same latent constructs across the three assessment occasions. For each scale and timepoint, a confirmatory factor analysis (CFA) was fitted using maximum likelihood estimation via the semopy package in Python. Model parameters were estimated analytically using the default maximum-likelihood standard errors; no bootstrap resampling was applied for the CFA parameter estimation. Bootstrap resampling (500 iterations) was used later, exclusively for the edge-stability analysis of the psychological networks (Section 2.2), and was not applied to the invariance CFAs. The DASS-21 was specified as a three-factor model with depression (items 3, 5, 10, 13, 16, 17, 21), anxiety (items 2, 4, 7, 9, 15, 19, 20), and stress (items 1, 6, 8, 11, 12, 14, 18) as latent factors. The CD-RISC-25 was specified as a unidimensional model with all 25 items loading on a single resilience factor.

Measurement invariance was evaluated using two complementary approaches. First, Tucker’s congruence coefficient (CC) was computed between the standardised factor loading vectors at each pair of timepoints. Following established conventions, CC values of.95 or above indicate factorial equality, values between.85 and.94 indicate fair similarity, and values below.85 indicate dissimilarity (28). Second, the delta-CFI criterion was applied, whereby a change in CFI of less than.01 between models supports measurement invariance (27). Standard model fit indices were reported including chi-square, CFI, TLI, RMSEA, and GFI.

### 2.3 Step 2: network analysis

Two psychological networks were estimated using graphical LASSO (least absolute shrinkage and selection operator) regularisation (29), which produces sparse partial correlation matrices by penalising small edges toward zero. This approach is now standard in psychological network analysis (23, 24) because it reduces the risk of false positive edges in small-sample, multi-variable settings. The regularisation parameter was selected via five-fold cross-validation using the GraphicalLassoCV implementation in scikit-learn (41). All variables were standardised prior to estimation.

Network A (cross-sectional) included 17 variables: Big Five personality traits (openness, conscientiousness, extraversion, agreeableness, neuroticism), DASS subscales at T0 (depression, anxiety, stress), resilience at T0, leadership, five OCB subscales (altruism, conscientiousness, sportsmanship, courtesy, civic virtue), and academic and athletic performance scores. Network B (temporal) included 12 variables: DASS depression, anxiety, and stress at T0, T1, and T2, and resilience at T0, T1, and T2. This network captures how distress components and resilience relate to each other across the training period.

Three centrality indices were computed for each node (42): strength (sum of absolute edge weights), betweenness (proportion of shortest paths passing through the node), and closeness (inverse of the average shortest path length to all other nodes). Edge stability was assessed using nonparametric bootstrap with 500 resampling iterations following the procedure of Epskamp et al. (2018) (24), computing the proportion of bootstraps in which each edge was non-zero (inclusion proportion). Edges with inclusion proportions above.90 were considered stable.

### 2.4 Step 3: latent profile analysis

Latent profile analysis (LPA) was conducted to identify distinct subgroups of cadets based on their baseline psychological characteristics (22, 43). The analysis employed Gaussian Mixture Models (GMM) with full covariance matrices, fitted using the expectation-maximisation algorithm (30). Ten baseline variables were included as profile indicators: five BFI personality traits (openness, conscientiousness, extraversion, agreeableness, neuroticism), three DASS-21 subscales at T0 (depression, anxiety, stress), resilience at T0 (CD-RISC-25), and leadership score. All variables were standardised prior to analysis.

Models with two through five profiles were evaluated. For each number of profiles, 20 random initialisations were conducted, each with five restarts, and the solution with the lowest Bayesian Information Criterion (BIC (44);) was retained. Model selection was based on the BIC (lower values indicating better fit), supplemented by the Akaike Information Criterion (AIC (45);), the Integrated Complete-data Likelihood (ICL (46);), classification entropy (higher values indicating clearer separation, with values above.80 considered acceptable (22);), silhouette score (47), and mean posterior classification probability.

The selected profile solution was characterised by computing descriptive statistics (means and standard deviations) for all study variables within each profile. Profile validation was conducted by testing whether the identified profiles differed significantly on outcome variables not used in profile identification, including OCB subscales, academic and athletic scores, and T1/T2 distress and resilience. Kruskal-Wallis H tests were used for continuous variables and chi-square tests for categorical variables, with Holm correction for multiple comparisons. Effect sizes were quantified using eta-squared for continuous variables and Cramer’s V for categorical variables.

#### 2.4.1 Sensitivity analysis: change-score latent profile analysis

To assess whether the latent profile solution is sensitive to the choice of measurement timepoint, a pre-specified supplementary LPA was conducted using the same Gaussian Mixture Model pipeline but substituting the T0 dynamic indicators (DASS depression, anxiety, stress, and CD-RISC resilience) with their T2 - T0 change scores. The trait-level inputs (five BFI dimensions and leadership) remained unchanged, preserving the ten-variable input dimensionality and therefore the statistical feasibility under the present sample size. Change scores were preferred over the inclusion of all three timepoints because raw multi-timepoint inclusion would have inflated the input space and introduced strong within-construct autoregressive dependence that GMM does not model. Concordance between the change-score and the primary T0-based profile assignments was quantified via the adjusted Rand index. Validation against T1 and T2 outcomes followed the same Kruskal-Wallis and chi-square procedure described above.

### 2.5 Step 4: risk nomograms

Clinical screening nomograms were developed using logistic regression to predict binary performance outcomes (high versus low, split at the median), following the methodological guidance of Iasonos et al. (2008) (25) and Steyerberg & Vergouwe (2014) (31). Three separate nomograms were constructed for OCB performance, academic performance, and athletic performance. Predictor variables were selected based on the results of prior classification analyses (37) and included baseline measures available at entry (T0), ensuring that the nomograms could serve as prospective screening tools.

All predictors were standardised prior to model fitting. Model coefficients were converted to odds ratios with approximate 95% confidence intervals. A point-based nomogram system was derived by scaling the absolute regression coefficients to a 0 to 100 range, where the predictor with the largest coefficient received 100 points (25). Predictive performance was evaluated using five-fold stratified cross-validation, reporting the area under the receiver operating characteristic curve (ROC-AUC) and the Brier score (48). Calibration was assessed by comparing predicted probabilities to observed outcome proportions across decile bins (26).

## 3 Results

### 3.1 Measurement invariance

**Figure 1:**
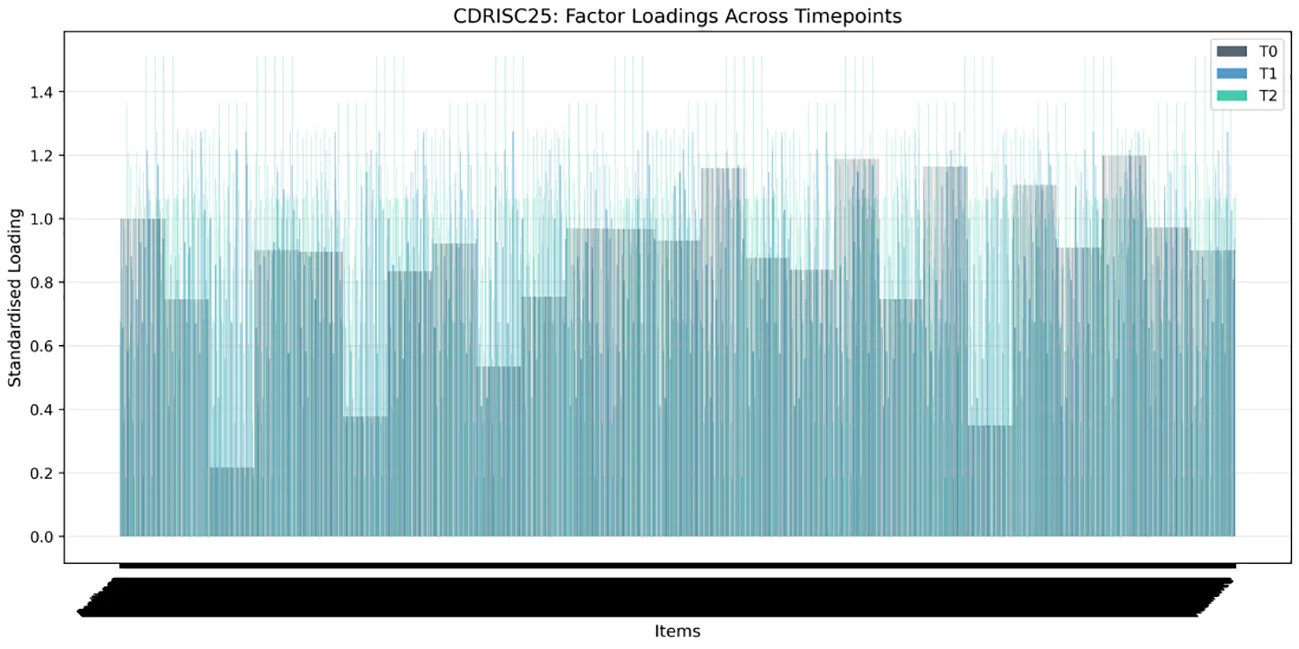
Standardised factor loadings of the CD-RISC-25 across T0, T1, and T2. Each bar represents the loading of an individual item on the resilience factor. The high consistency across timepoints (all congruence coefficients above.95) indicates that the items contribute equivalently to the resilience construct regardless of the measurement occasion.

The CD-RISC-25 demonstrated excellent factorial stability across all three timepoints. Tucker’s congruence coefficients were.984 for T0 versus T1,.970 for T0 versus T2, and.958 for T1 versus T2, yielding a mean congruence of.971 (**Table 1**, **Figures 1**, **3**). All values exceeded the.95 threshold for factorial equality, indicating that the unidimensional resilience factor structure was maintained throughout the training period.

**Figure 2:**
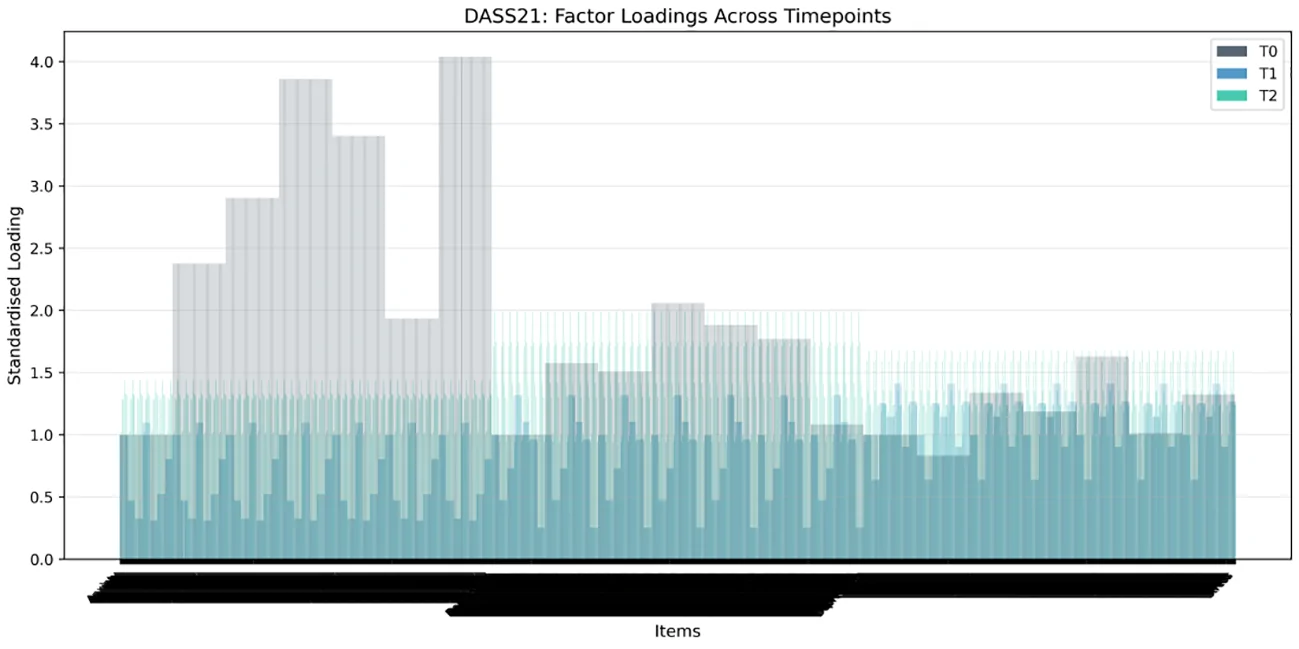
Standardised factor loadings of the DASS-21 three-factor model across T0, T1, and T2. The first seven items correspond to the depression subscale, items 8 through 14 to the anxiety subscale, and items 15 through 21 to the stress subscale. Some loading variation is visible at T1, consistent with the lower T0 versus T1 congruence coefficient (.790).

The DASS-21 showed fair factorial stability. Congruence coefficients were.790 for T0 versus T1,.887 for T0 versus T2, and.924 for T1 versus T2, yielding a mean congruence of.867. While the T0 versus T1 comparison fell slightly below the.85 fair similarity threshold, the overall mean supported adequate invariance (**Table 1**, **Figure 2**). The lower congruence at T0 versus T1 may reflect genuine shifts in distress factor structure during the acute stress of basic training, which stabilised by T2 .

**Table 1:** Tucker’s congruence coefficients for factor loadings across timepoints.

Scale	CC T0-T1	CC T0-T2	CC T1-T2	Mean CC	Interpretation
CD-RISC-25	.984	.970	.958	.971	Excellent
DASS-21	.790	.887	.924	.867	Fair

Values above .95 indicate factorial equality, values between .85 and .94 indicate fair similarity.

**Figure 3:**
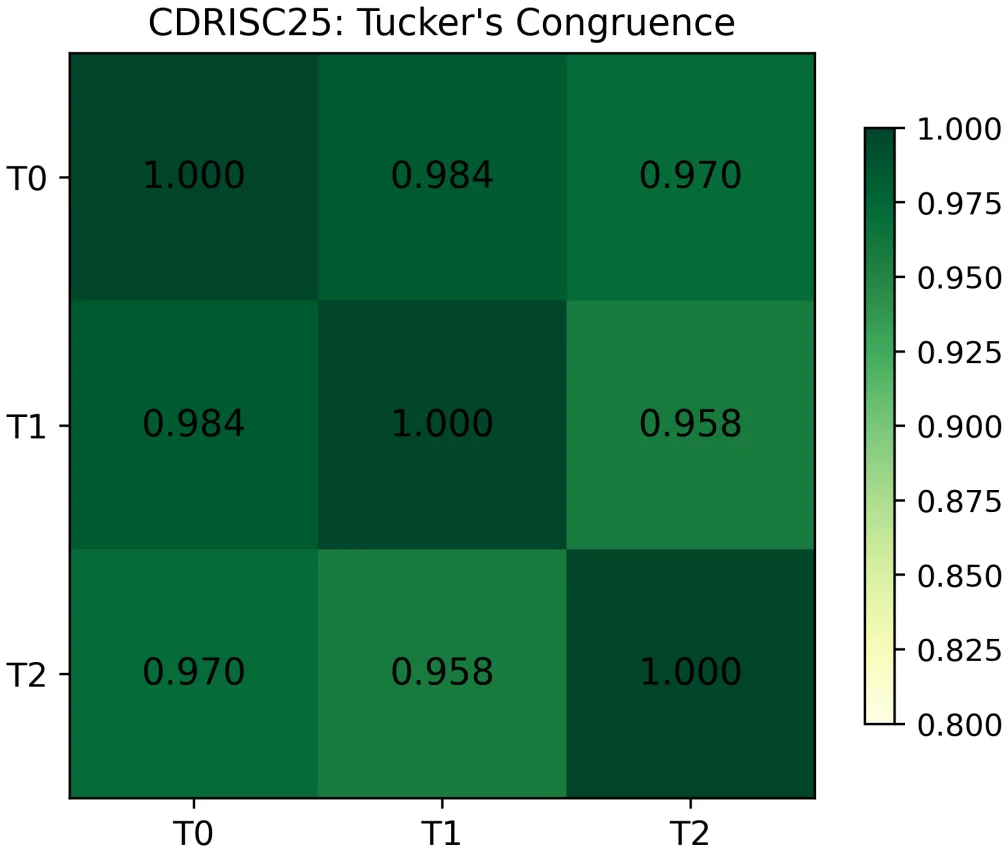
Heatmap of Tucker’s congruence coefficients for the CD-RISC-25. All pairwise comparisons exceed the.95 criterion for factorial equality, confirming measurement invariance.

### 3.2 Network analysis

#### 3.2.1 Cross-sectional network

**Figure 4:**
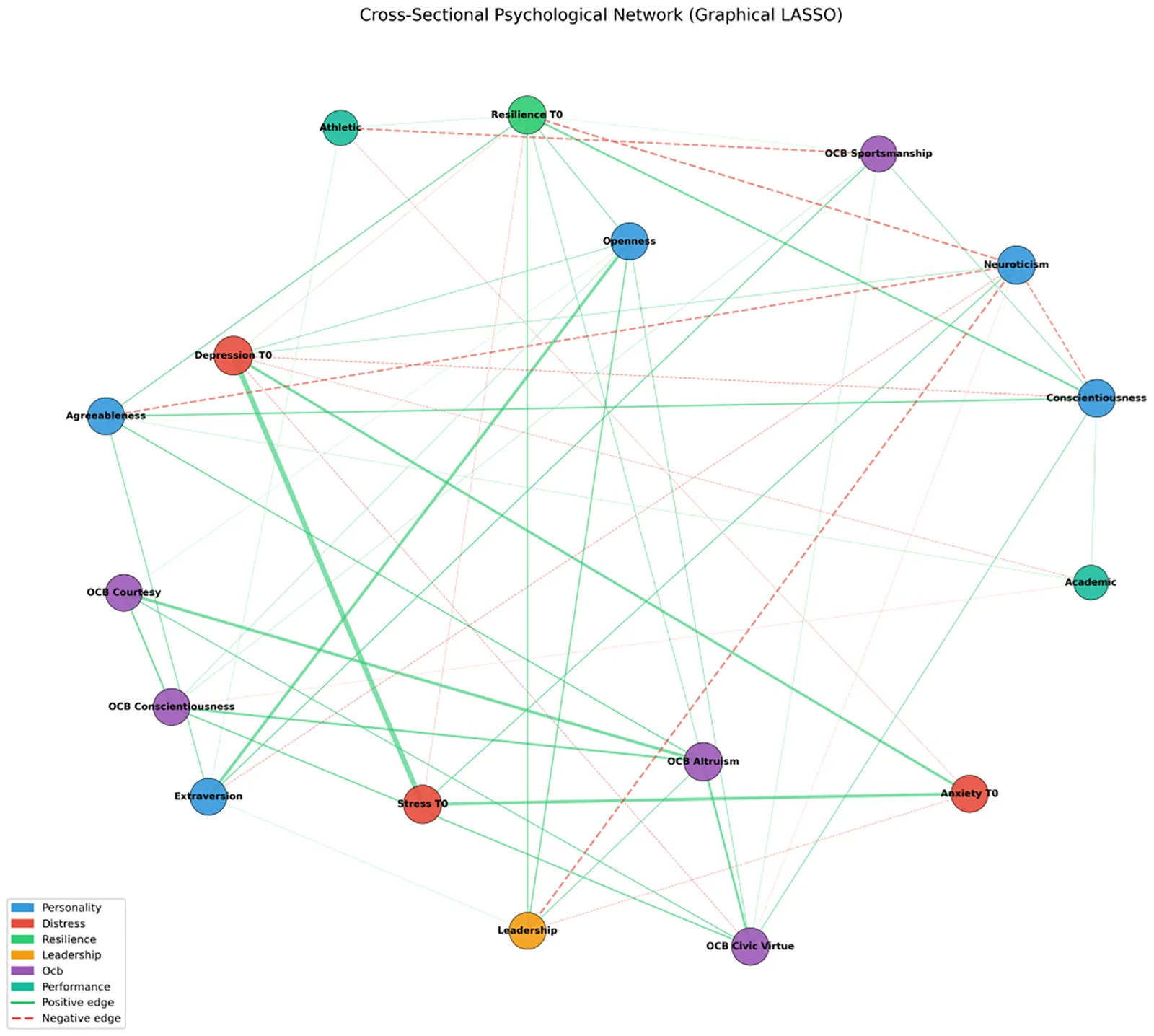
Cross-sectional psychological network estimated using graphical LASSO. Nodes are coloured by domain: blue represents personality traits, red represents distress symptoms, green represents resilience, yellow represents leadership, purple represents OCB subscales, and teal represents performance outcomes. Solid green edges indicate positive partial correlations and dashed red edges indicate negative partial correlations. Node size is proportional to strength centrality. The prominent cluster linking depression, anxiety, and stress reflects their shared variance as distress indicators, while resilience bridges this cluster to the personality domain.

The cross-sectional network (**Figure 4** Network A) revealed a well-connected structure with 52 edges exceeding the absolute partial correlation threshold of.02. The strongest edge in the network was between depression at T0 and stress at T0 (partial r = .541, bootstrap inclusion = 100%), followed by anxiety and stress (.339, 100%), OCB altruism and OCB courtesy (.330, 100%), and openness and extraversion (.321, 99.6%). The depression and stress coupling was notably robust, appearing in every bootstrap sample (**Figure 6**).

**Figure 5:**
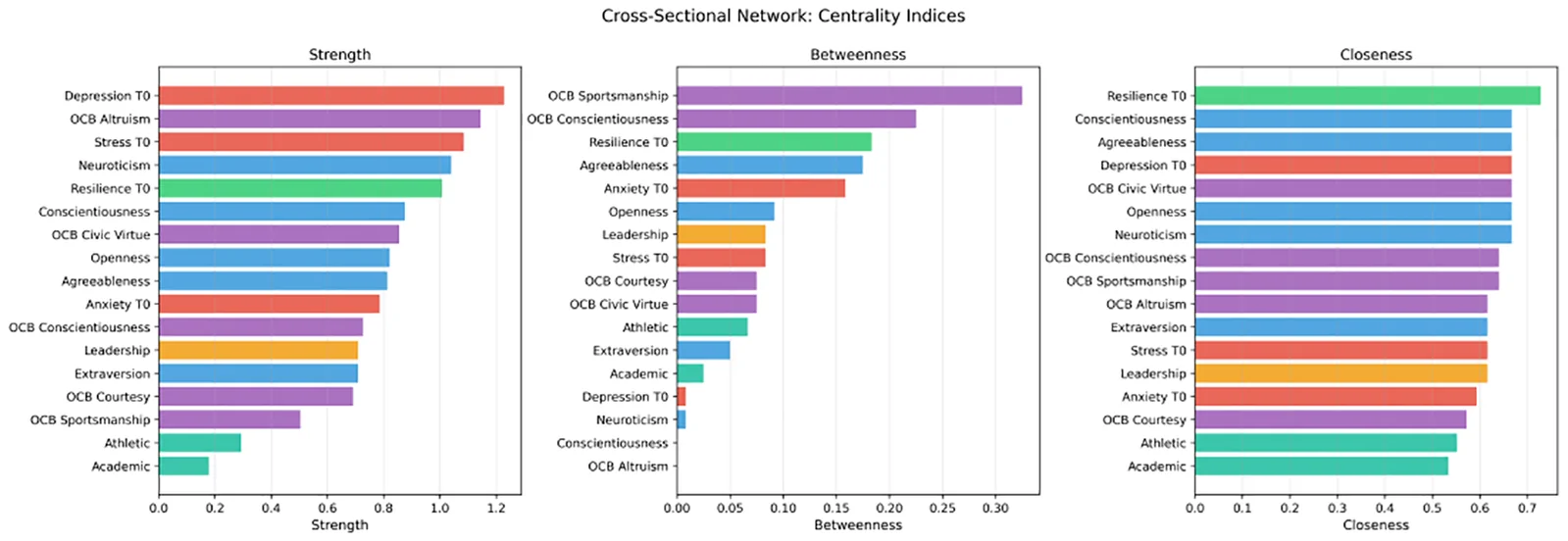
Centrality indices (strength, betweenness, closeness) for all nodes in the cross-sectional network. Depression T0 and OCB Altruism exhibit the highest strength, while Resilience T0 and OCB Sportsmanship show the highest betweenness, indicating their role as bridge nodes connecting different psychological domains.

Centrality analysis identified depression at T0 as the node with the highest strength (**Table 2**, **Figure 5**) (1.228), followed by OCB altruism (1.144) and stress at T0 (1.085). Resilience at T0 exhibited the highest betweenness centrality (.183), indicating that it served as a bridging node connecting the distress cluster to the personality and OCB domains. This finding positions resilience as a potential intervention target, as changes in resilience may propagate across both distress reduction and enhanced organisational behaviour.

**Table 2:** Centrality indices for the top nine nodes in the cross-sectional psychological network.

Node	Strength	Betweenness	Closeness
Depression T0	1.228	0.008	0.667
OCB Altruism	1.144	0.000	0.615
Stress T0	1.085	0.083	0.615
Neuroticism	1.040	0.008	0.667
Resilience T0	1.007	0.183	0.727
Conscientiousness	0.875	0.000	0.667
OCB Civic Virtue	0.855	0.075	0.667
Openness	0.821	0.092	0.667
Agreeableness	0.813	0.175	0.667

Strength represents the sum of absolute edge weights, betweenness represents the proportion of shortest paths traversing the node, and closeness represents the inverse average distance to all other nodes.

**Figure 6:**
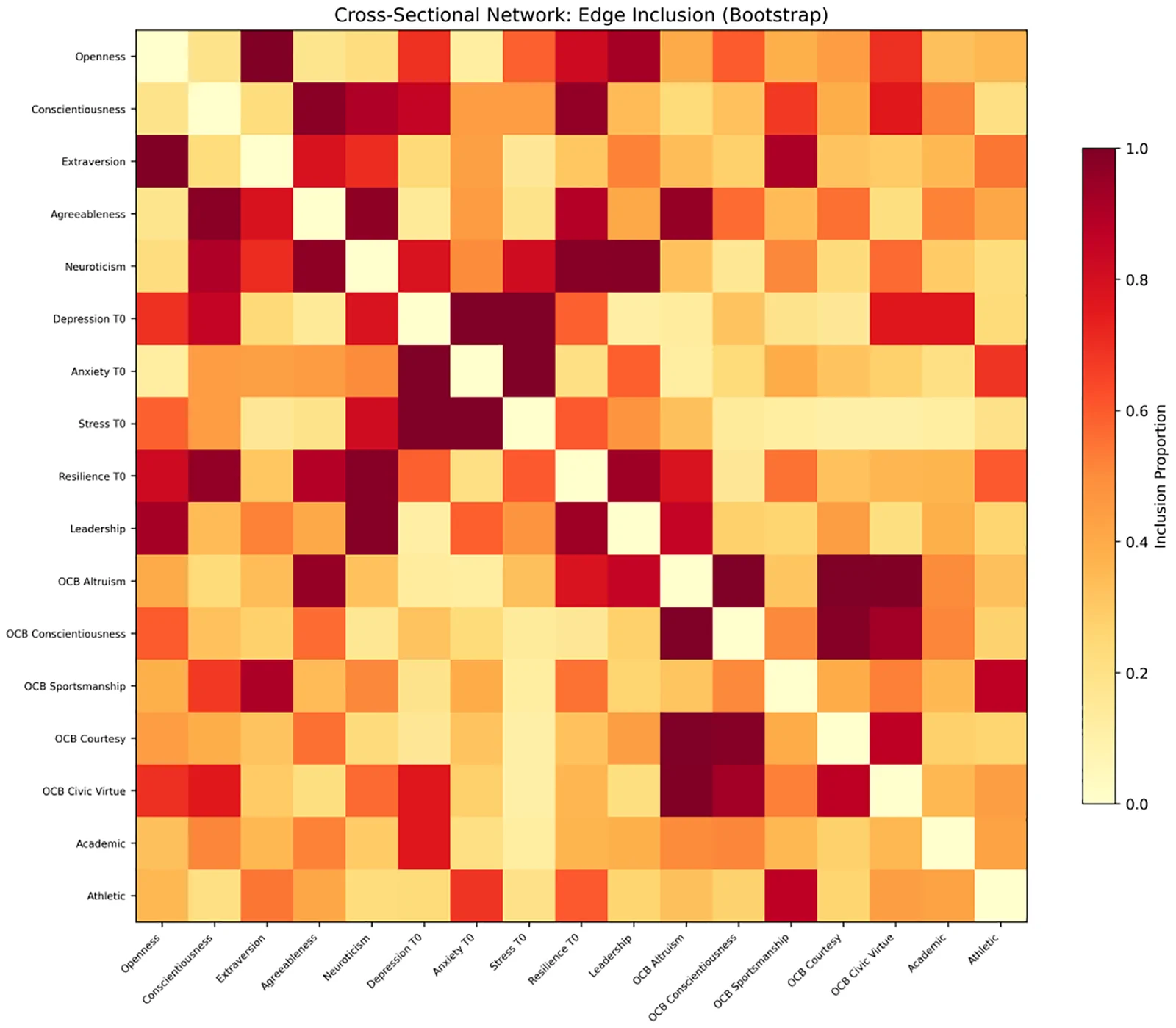
Bootstrap edge stability heatmap for the cross-sectional network (500 iterations). Darker cells indicate higher inclusion proportions, reflecting greater stability. The depression-stress and anxiety-stress edges achieved 100% inclusion, confirming their robustness.

#### 3.2.2 Temporal network

**Figure 7:**
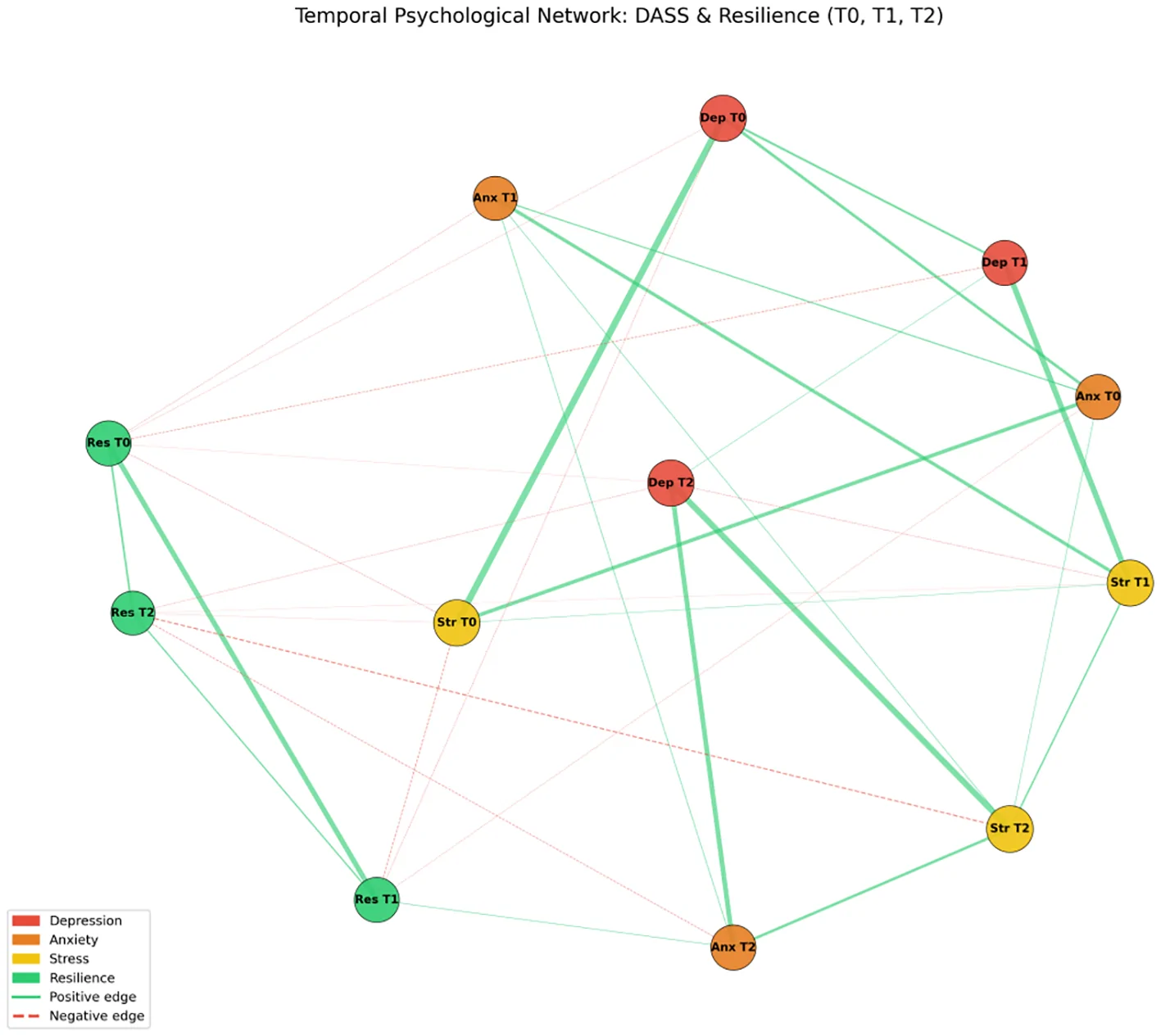
Temporal psychological network showing DASS subscales (depression, anxiety, stress) and resilience across T0, T1, and T2. Node colours distinguish depression (red), anxiety (orange), stress (yellow), and resilience (green). The strong within-timepoint clustering of distress components is visible, alongside cross-timepoint resilience stability.

**Figure 8:**
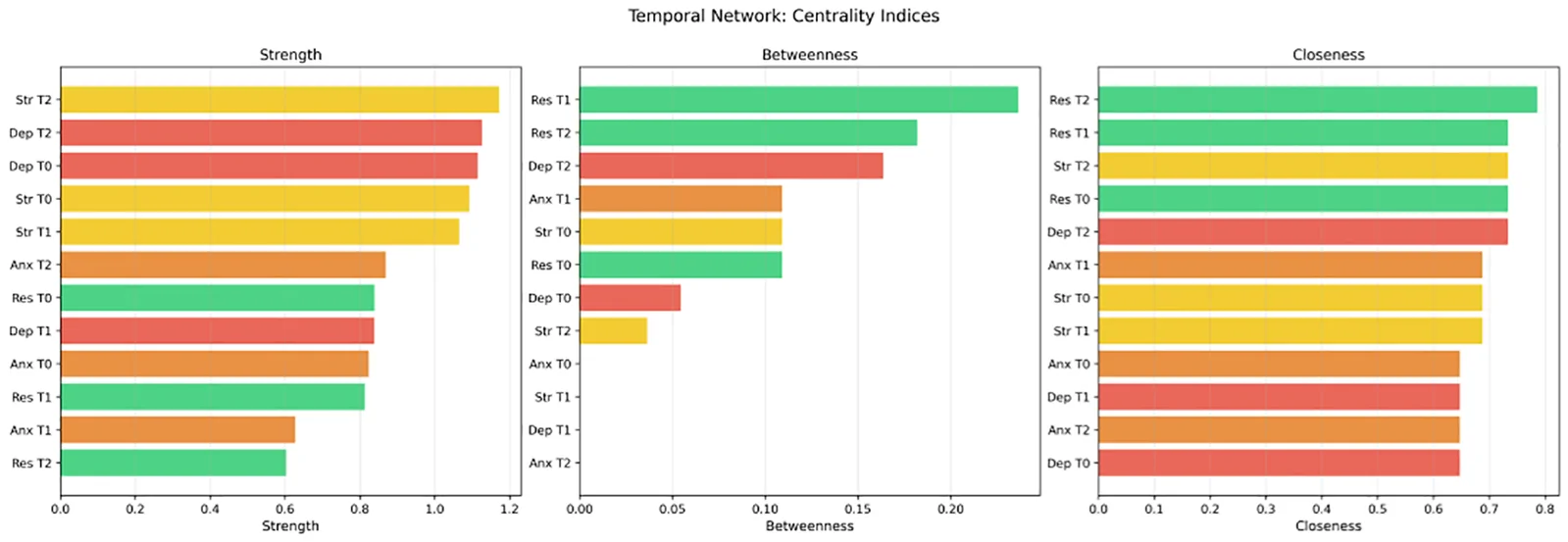
Centrality indices for the temporal network. Stress at T2 shows the highest strength, while Resilience T1 has the highest betweenness, suggesting it acts as a temporal bridge between early and late measurements.

The temporal network (Network B, **Figures 7**, **8**) captured the dynamic relationships among distress and resilience across three timepoints. The strongest edges were within-construct, same-timepoint associations: depression and stress at T0 (partial r = .567), at T2 (.517), and at T1 (.488). These within-timepoint couplings were remarkably stable, suggesting that the co-occurrence of depression and stress is a robust structural feature rather than a transient phenomenon.

Cross-timepoint edges revealed that resilience at T0 was strongly connected to resilience at T1 (partial r = .445), supporting temporal stability of individual differences in resilience. Depression at T0 showed a moderate connection to depression at T1 (.202), while the corresponding anxiety and stress carry-over effects were weaker after accounting for other variables. Resilience at T2 exhibited the highest closeness centrality (.786), indicating that end-of-semester resilience was the most efficiently connected node in the temporal network, reachable through short paths from all other variables.

### 3.3 Latent profile analysis

Model comparison across two through five profile solutions indicated that the five-profile model provided the best fit, achieving the lowest BIC (2277.4) (**Table 3**). Classification quality was excellent, with an entropy of.973 and a mean posterior classification probability of.986, indicating that cadets were assigned to their most likely profile with high certainty.

**Table 3:** Model comparison for latent profile analysis solutions with two through five profiles.

Profiles (k)	AIC	BIC	ICL	Entropy	Silhouette	Smallest class
2	2176.8	2507.2	2519.6	.903	.239	36 (39.1%)
3	1971.0	2467.8	2474.6	.966	.199	3 (3.3%)
4	1768.2	2431.5	2435.6	.984	.181	5 (5.4%)
5	1447.7	2277.4	2285.3	.973	.101	4 (4.3%)

The five-profile solution was selected based on the lowest BIC. Entropy values above.80 indicate acceptable classification quality.

The five profiles were characterised as follows. Profile 1 (n = 26, 28.3%) represented a moderate adaptation group with average scores across most indicators. Profile 2 (n = 46, 50.0%) comprised the largest group, characterised by a well-adjusted pattern with moderate resilience and low distress. Profile 3 (n = 4, 4.3%) was a small group with extreme scores, likely representing cadets with distinctive psychological profiles that warranted individual attention. Profile 4 (n = 10, 10.9%) showed elevated distress combined with lower resilience, suggesting a vulnerable subgroup. Profile 5 (n = 6, 6.5%) exhibited a distinctive pattern combining specific personality and distress characteristics.

**Figure 9:**
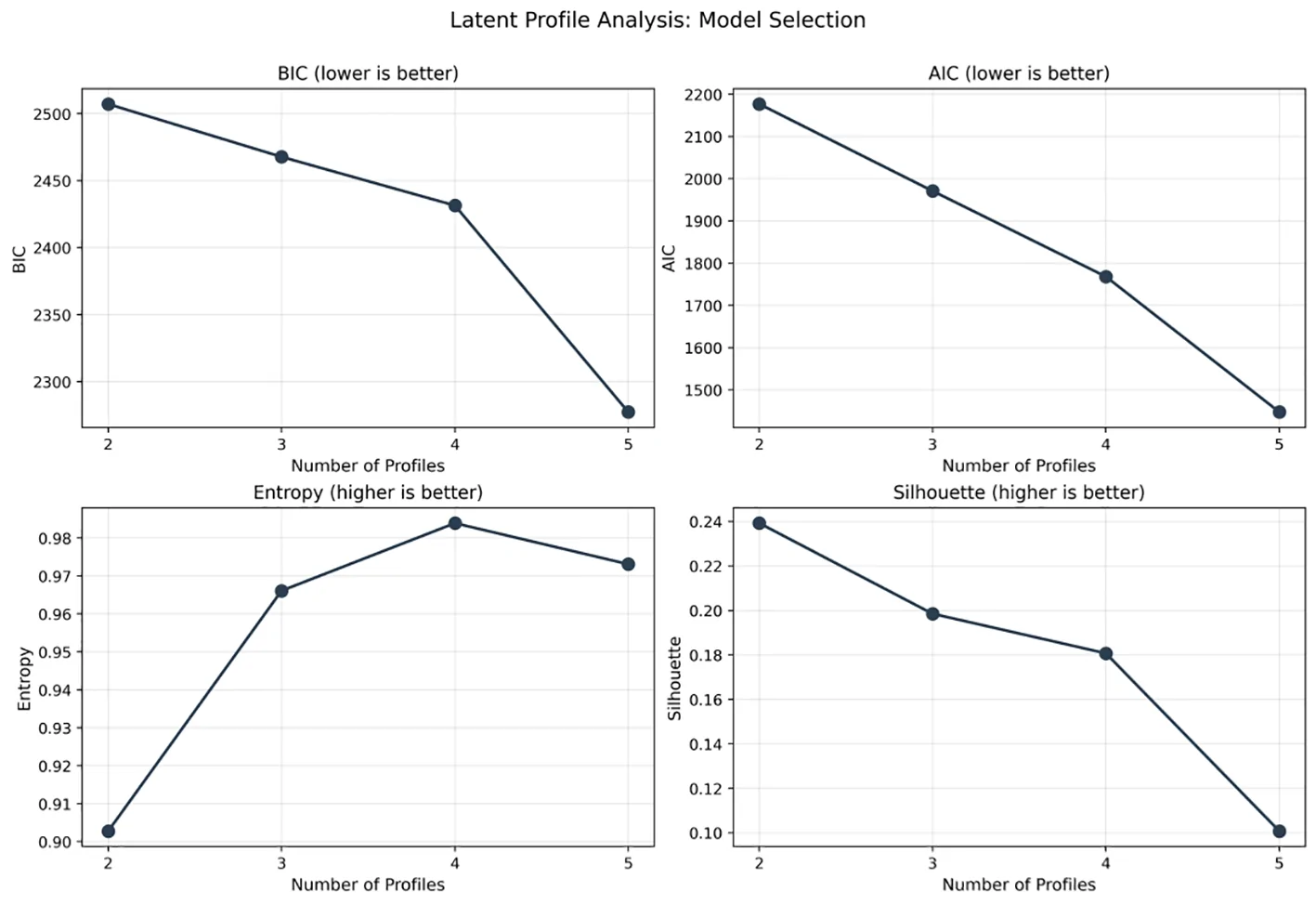
Model selection criteria across profile solutions (k = 2 to 5). The upper left panel shows BIC values (lower is better), indicating that the five-profile solution achieved the best fit. The upper right panel shows AIC, which similarly favoured five profiles. Entropy (lower left) remained high across all solutions, and the silhouette score (lower right) decreased as more profiles were extracted, reflecting the typical tradeoff between profile granularity and within-profile homogeneity.

Validation analyses confirmed that the five profiles differed significantly on 17 of the continuous variables examined after Holm correction. The largest effect sizes were observed for depression at T0 (eta-squared = .605), stress at T0 (.497), anxiety at T0 (.453), neuroticism (.358), and conscientiousness (.250). These results indicate that the profiles captured meaningful individual differences that extended to outcomes not used in profile identification, including OCB score (eta-squared = .144), athletic performance (.142), and resilience at T2 (.146 ). **Figure 9** depicts the Model selection criteria across profile solutions (k = 2 to 5). The upper left panel shows BIC values (lower is better), indicating that the five-profile solution achieved the best fit. The upper right panel shows AIC, which similarly favoured five profiles. Entropy (lower left) remained high across all solutions, and the silhouette score (lower right) decreased as more profiles were extracted, reflecting the typical tradeoff between profile granularity and within-profile homogeneity.

**Table 4:** Kruskal-Wallis H tests comparing the five latent profiles on continuous variables.

Variable	H	p (Holm)	eta-sq	Significant
dass_depression_t0	56.60	<.001	.605	Yes
dass_stress_t0	47.27	<.001	.497	Yes
dass_anxiety_t0	43.45	<.001	.453	Yes
bfi_neuroticism	35.13	<.001	.358	Yes
cdrisc_score_t0	28.39	<.001	.280	Yes
bfi_conscientiousness	25.73	<.001	.250	Yes
dass_depression_t1	25.24	<.001	.244	Yes
bfi_extraversion	19.51	.012	.178	Yes
bfi_openness	17.95	.021	.160	Yes
leadership_score	18.42	.018	.166	Yes
ocb_score	16.55	.033	.144	Yes
cdrisc_score_t2	16.71	.033	.146	Yes
ath_score	16.36	.033	.142	Yes

Only significant results (p <.05 after Holm correction) are shown. Effect sizes are reported as eta-squared.

**Figure 10:**
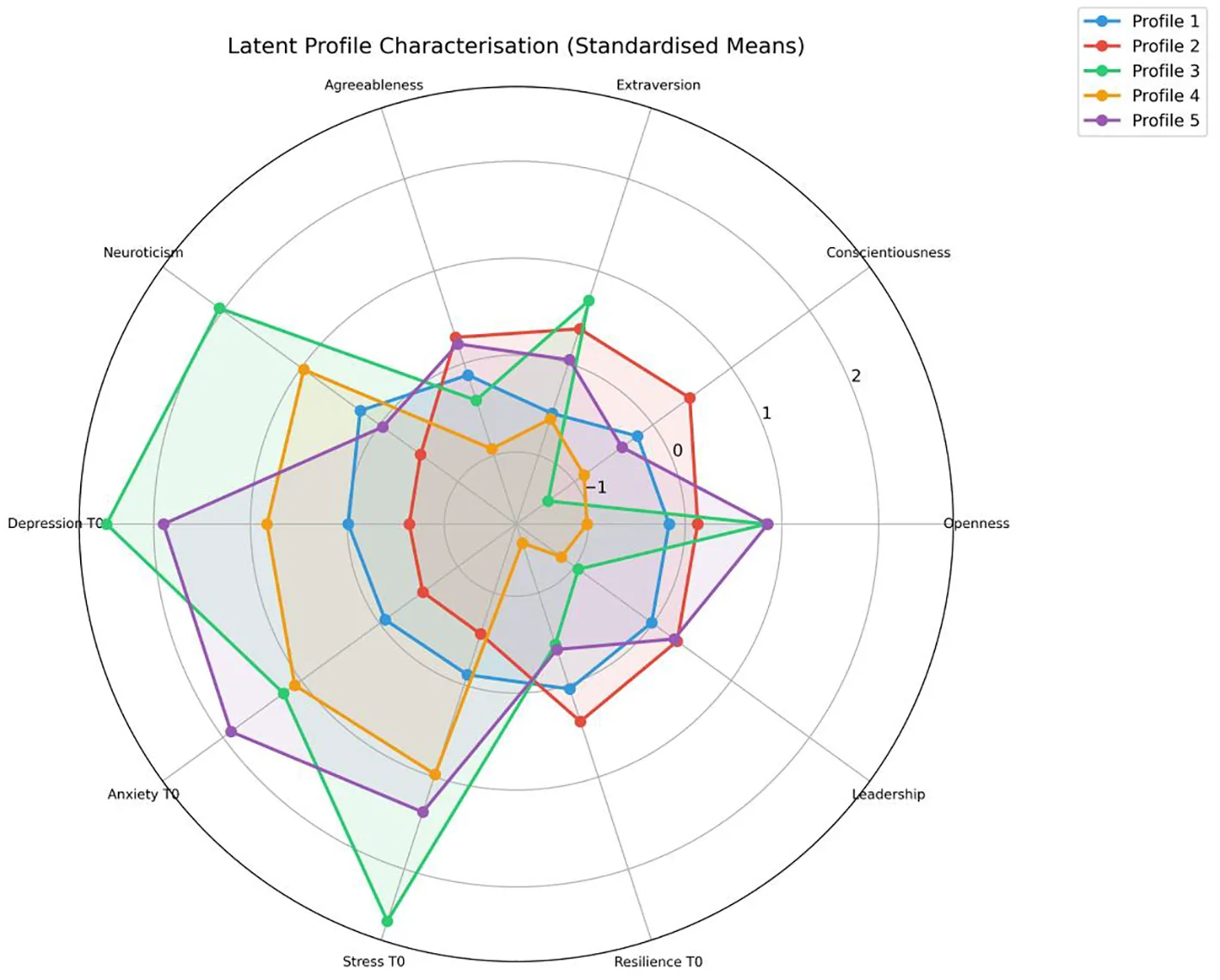
Radar plot of standardised mean scores for each latent profile across the ten indicator variables. Each axis represents a standardised variable (z-score), where values above zero indicate above-average scores and values below zero indicate below-average scores relative to the full sample. The distinct angular patterns confirm qualitative differences between profiles.

**Figure 11:**
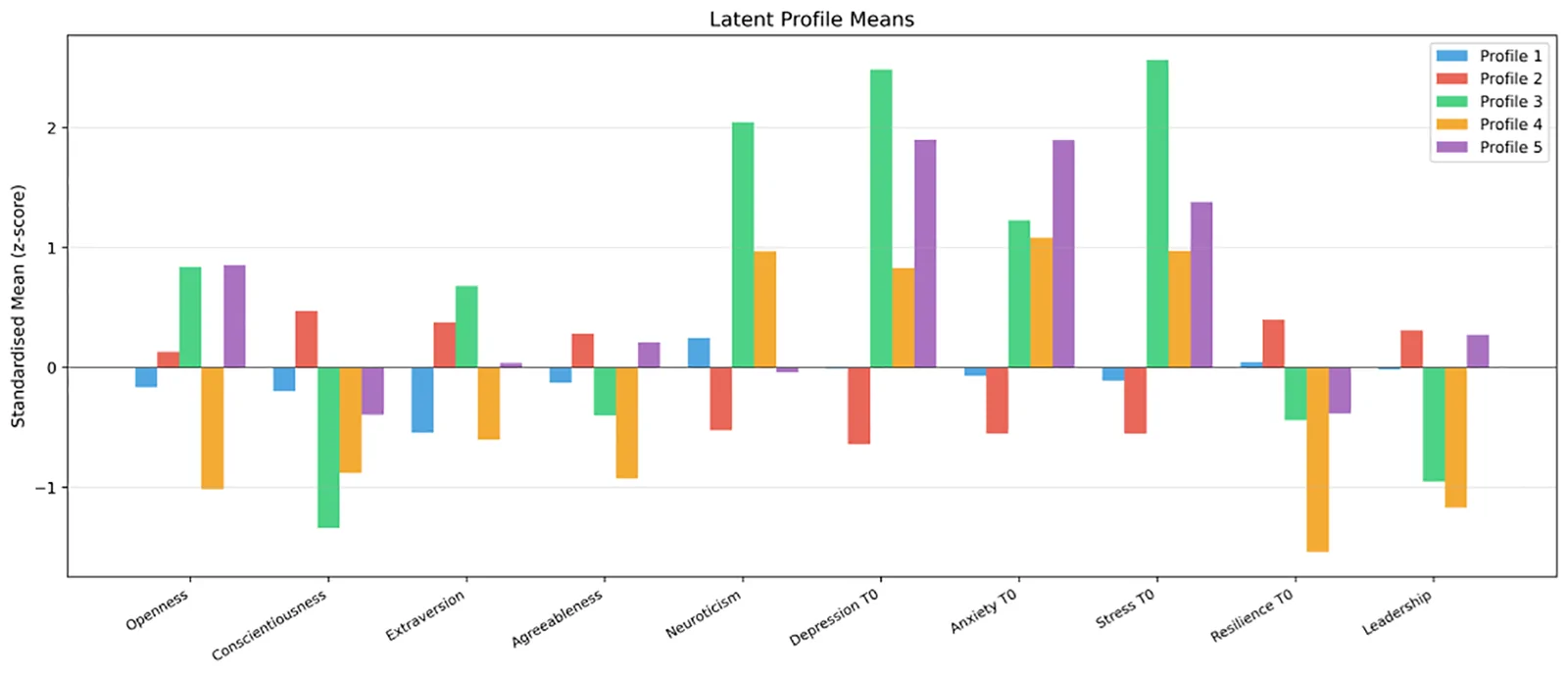
Grouped bar chart of standardised profile means across indicator variables. This representation complements the radar plot by providing a linear comparison of profile differences on each variable.

**Figure 12:**
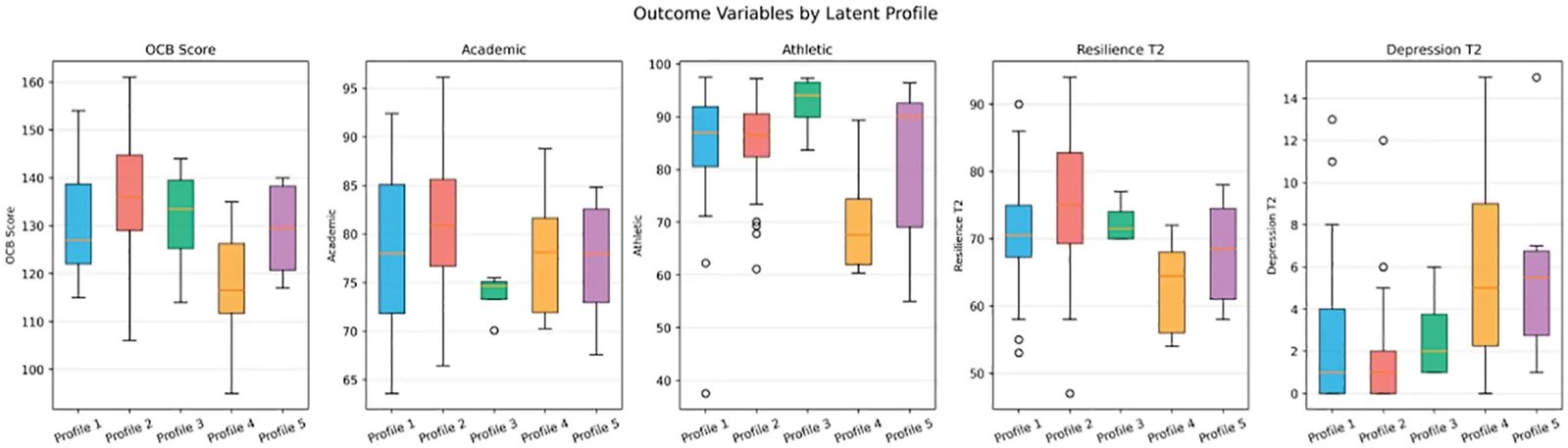
Box plots of key outcome variables by latent profile. These outcomes were not used in profile identification, serving as external validation criteria. Differences in OCB score, academic performance, athletic performance, resilience at T2, and depression at T2 confirm the predictive validity of the profile solution.

### 3.4 Sensitivity analysis: change-score LPA

The change-score sensitivity analysis was conducted to evaluate the robustness of the profile typology to the choice of measurement timepoint. Model comparison again favoured the five-profile solution by BIC (2409.0), with high classification entropy (.955), high mean posterior classification probability (.971), and class sizes of 7, 44, 30, 3, and 8 cadets. Validation analyses revealed fourteen continuous variables that differed significantly across the change-score profiles after Holm correction, with the largest effect sizes observed for the change-score inputs themselves (delta-depression eta-squared = .589; delta-stress.497; delta-anxiety.317; delta-resilience.138) together with personality traits (conscientiousness.269; agreeableness.249; extraversion.203; neuroticism.190) and concurrent T2 distress (depression T2.213, anxiety T2.182, stress T2.183). Importantly, neither academic, athletic, nor OCB outcomes reached significance after Holm correction in the change-score solution, indicating that trajectories of distress and resilience capture a dimension of variability that does not map directly onto external performance outcomes. **Table 4** presetnes the results of Kruskal-Wallis H tests comparing the five latent profiles on continuous variables, while **Table 5** shows the model comparison for the sensitivity (change-score) latent profile analysis. **Figures 10** and **11** illustrate the Radar plot, and grouped bar chart of standardised mean scores for each latent profile across indicator variables, respectively, while the **Figure 12** shows the Box plots of key outcome variables by latent profile.

**Table 5:** Model comparison for the sensitivity (change-score) latent profile analysis.

Profiles (k)	AIC	BIC	ICL	Entropy	Silhouette	Smallest class
2	2143.4	2473.8	2473.8	1.000	.196	8 (8.7%)
3	2041.3	2538.1	2553.4	.925	.137	6 (6.5%)
4	1763.0	2426.2	2429.8	.986	.076	4 (4.3%)
5	1579.3	2409.0	2422.2	.955	.091	3 (3.3%)

The five-profile solution was retained for parity with the primary analysis and because it minimised BIC.

**Figure 13:**
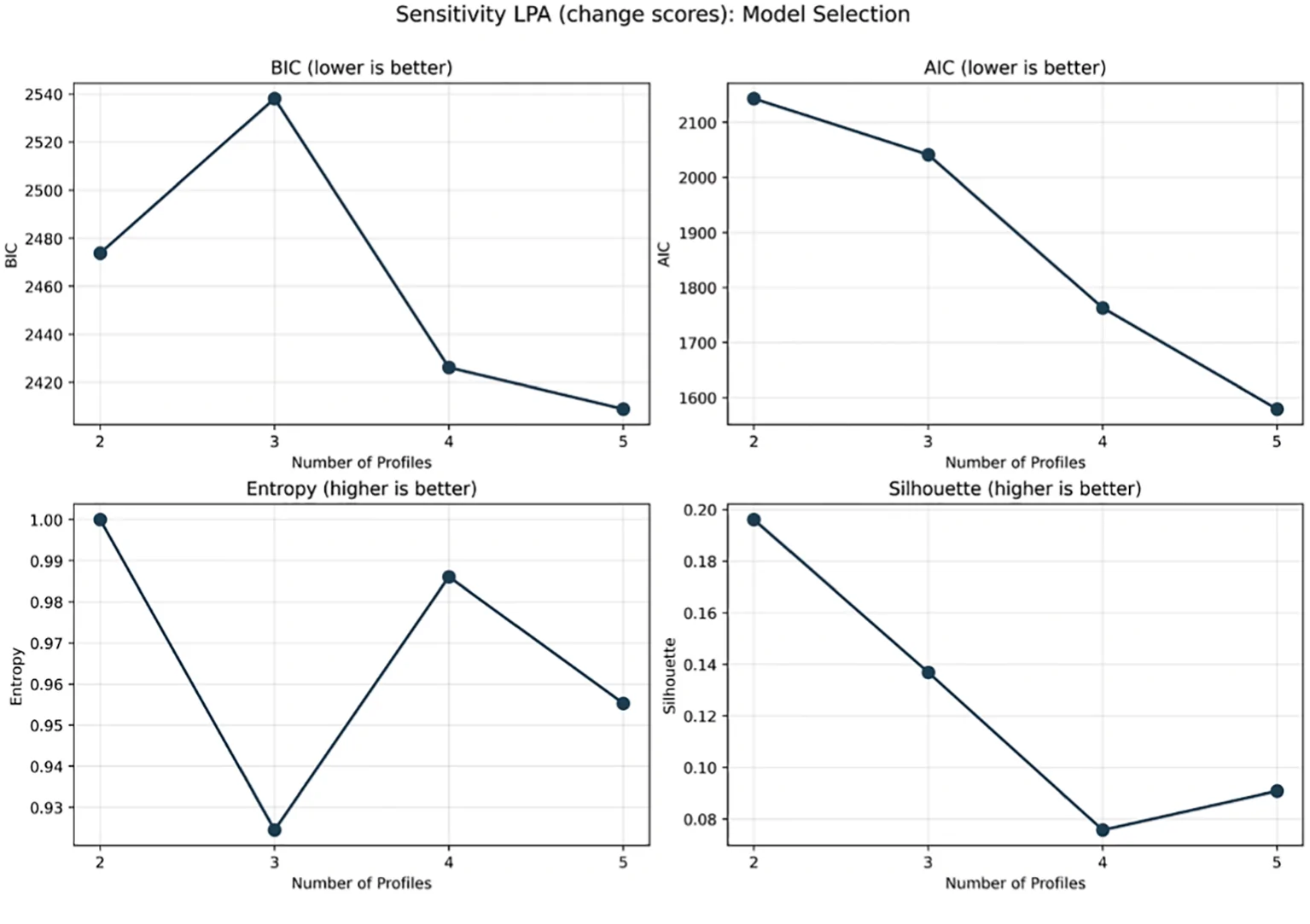
Model selection criteria for the change-score sensitivity LPA across two through five profile solutions. The five-profile solution achieved the lowest BIC and the highest mean posterior probability.

**Figure 14:**
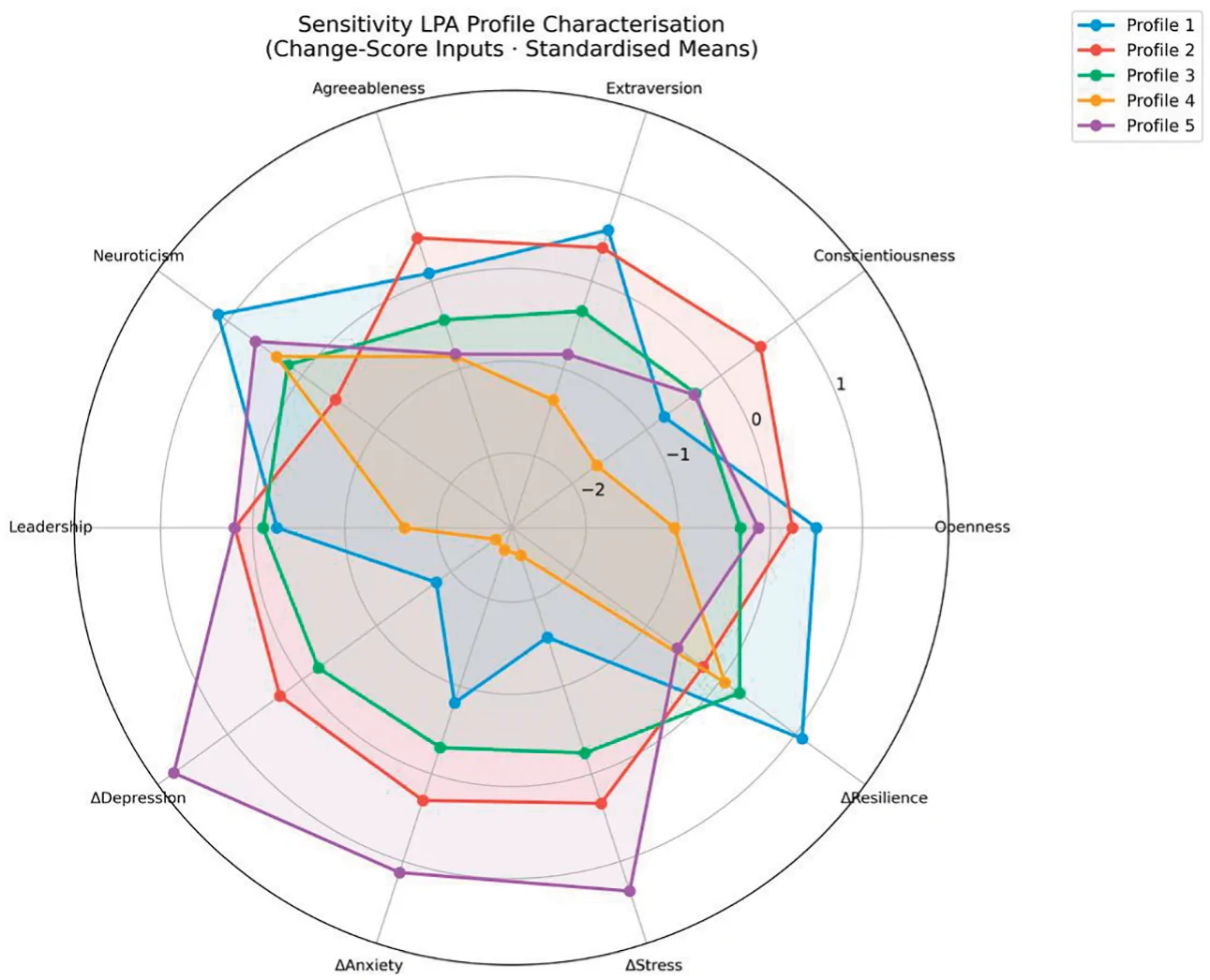
Radar plot of standardised mean scores for each change-score sensitivity profile. The dominant axes of differentiation are the four change-score indicators (delta-depression, delta-anxiety, delta-stress, delta-resilience), reflecting the substantive shift from a baseline-state typology to a trajectory typology.

**Figure 15:**
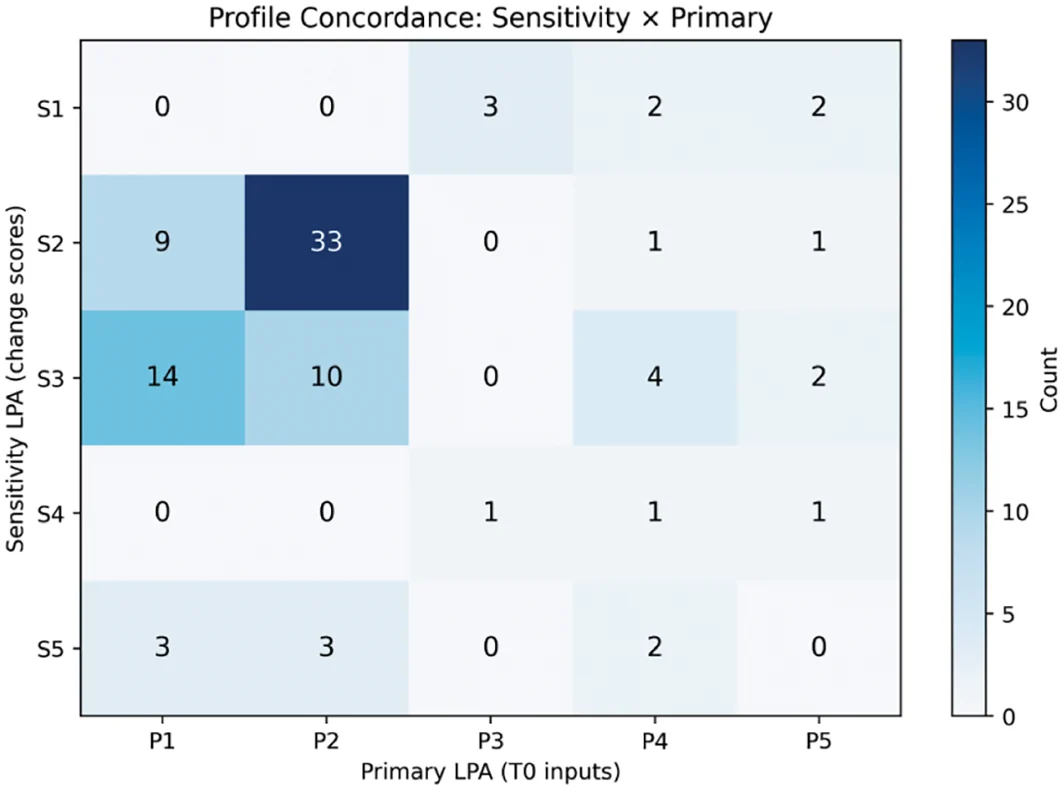
Cross-tabulation heatmap of profile assignments under the primary T0-based LPA (columns) versus the sensitivity change-score LPA (rows). The adjusted Rand index of.245 reflects the moderate concordance between the two solutions, supporting the interpretation that baseline configuration and longitudinal trajectory are complementary aspects of cadet psychological heterogeneity.

Concordance between the sensitivity and primary profile assignments was modest (**Figures 13**–**15**). The adjusted Rand index was.245, indicating that approximately one quarter of the pairwise agreement structure was shared between the two solutions. The cross-tabulation showed that primary profile 2 (the largest, well-adjusted group at baseline) distributed mainly into sensitivity profiles 2 (n = 33) and 3 (n = 10), whereas primary profile 1 (the moderate-adaptation group) split across sensitivity profiles 2 (n = 9) and 3 (n = 14). The small extreme-score primary profiles 3, 4, and 5 redistributed across all five sensitivity profiles. Taken together, these results suggest that the two LPA solutions characterise complementary rather than redundant facets of cadet heterogeneity: the primary T0 analysis identifies cadets by their baseline psychological configuration, while the change-score analysis identifies cadets by the direction and magnitude of their distress and resilience trajectories across the training period.

### 3.5 Risk nomograms

Three logistic regression nomograms were developed to predict high versus low performance using baseline measures (**Table 6**). The academic performance nomogram achieved the highest cross-validated predictive accuracy (AUC = .682, Brier score = .232), followed by the OCB nomogram (AUC = .544, Brier = .277) and the athletic nomogram (AUC = .424, Brier = . 5292 ).

**Table 6:** Summary of logistic regression nomogram performance.

Target	AUC (CV)	Brier score	Strongest predictor	OR
Academic	.682	.232	Gender	2.77
OCB	.544	.277	Depression T0	0.58
Athletic	.424	.292	Extraversion	1.39

AUC was computed via five-fold stratified cross-validation. OR represents the odds ratio per standard deviation increase in the strongest predictor.

**Figure 16:**
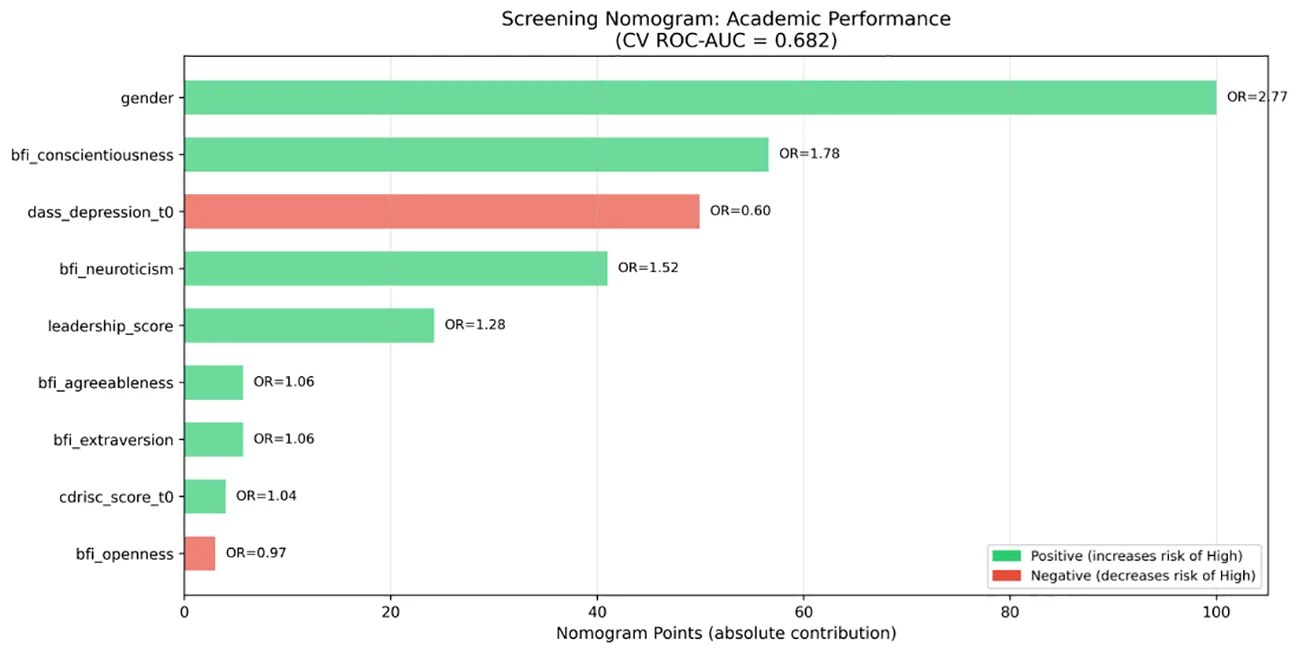
Screening nomogram for academic performance. Each bar represents the absolute point contribution of a predictor, scaled so that the strongest predictor receives 100 points. Green bars indicate positive predictors (higher values increase the probability of high academic performance), while red bars indicate negative predictors. Odds ratios are annotated for each predictor.

**Figure 17:**
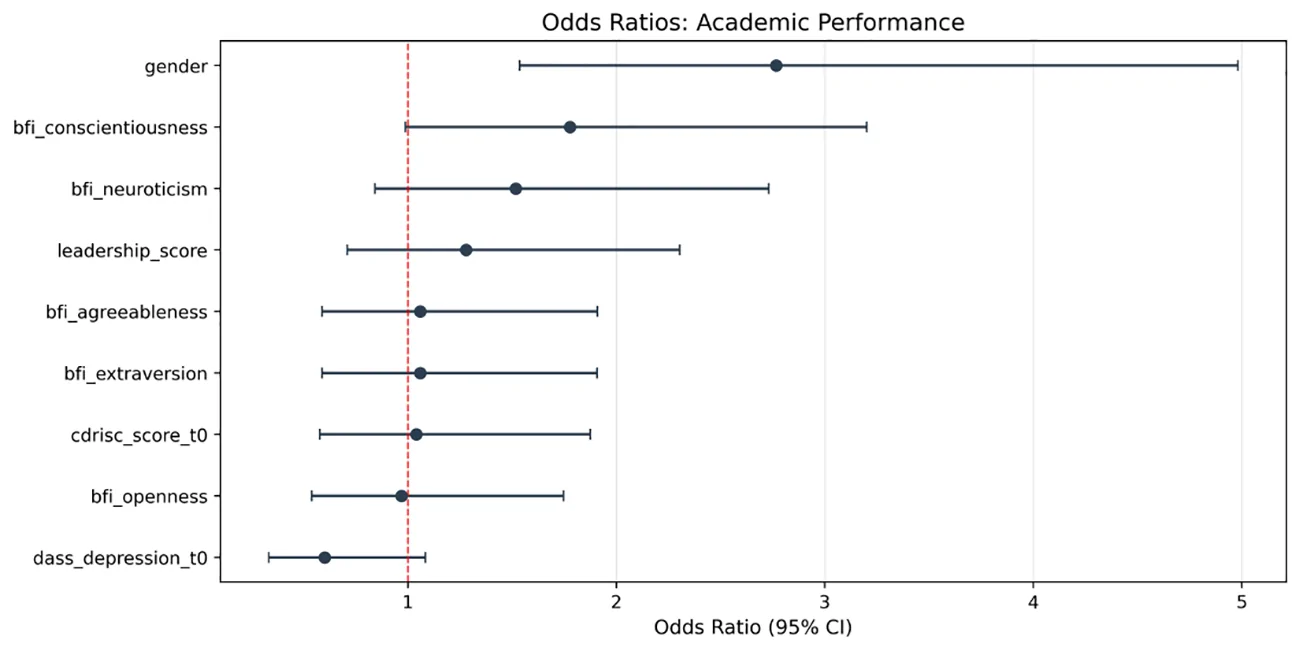
Forest plot of odds ratios with 95% confidence intervals for the academic performance model. The vertical dashed line at OR = 1 represents no effect. Predictors whose confidence intervals do not cross this line demonstrate statistically meaningful contributions.

**Figure 18:**
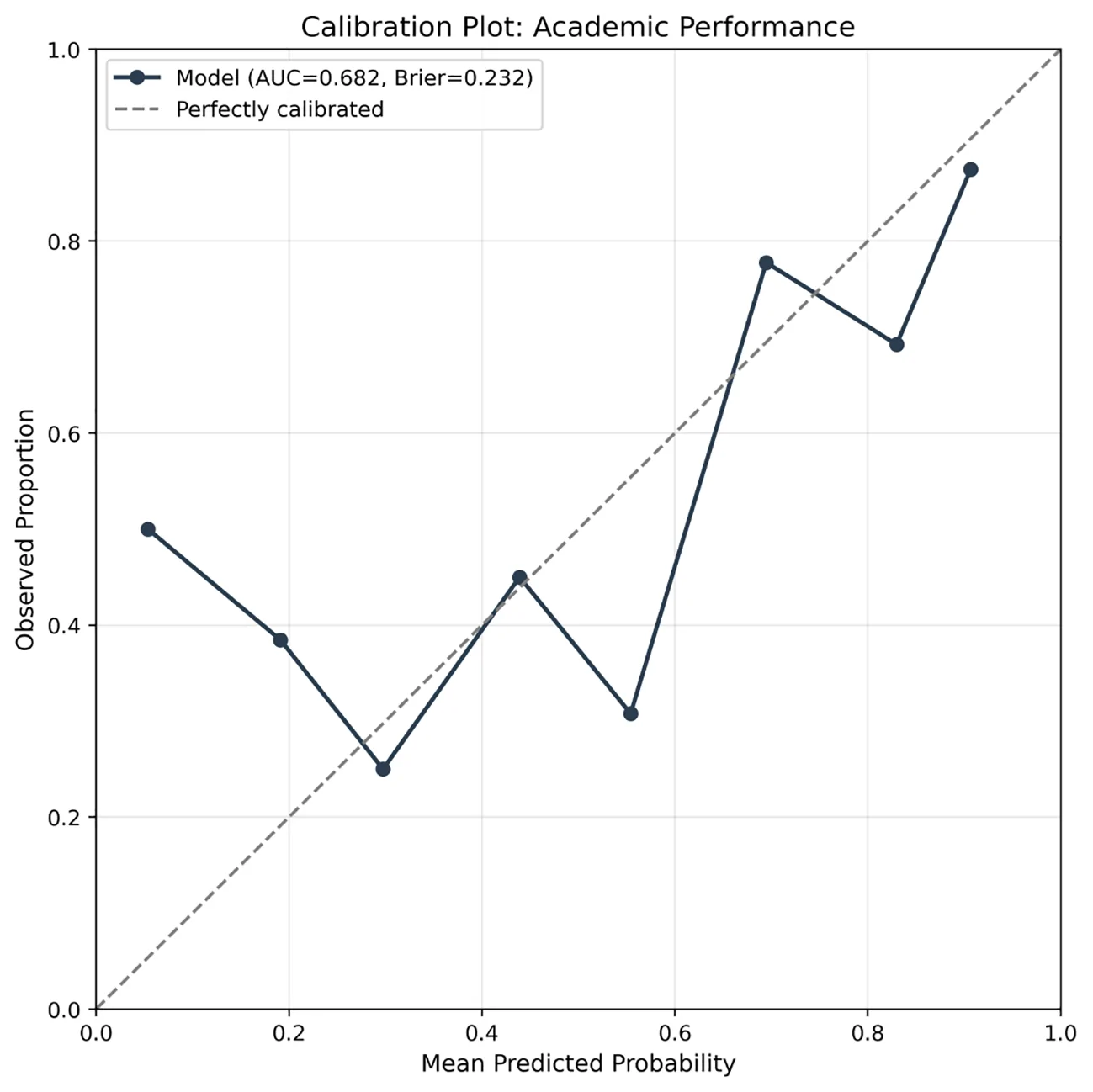
Calibration plot for the academic performance nomogram. The horizontal axis represents the mean predicted probability within each decile bin, and the vertical axis represents the observed proportion of high-performance cadets. Proximity to the diagonal indicates good calibration.

**Figure 19:**
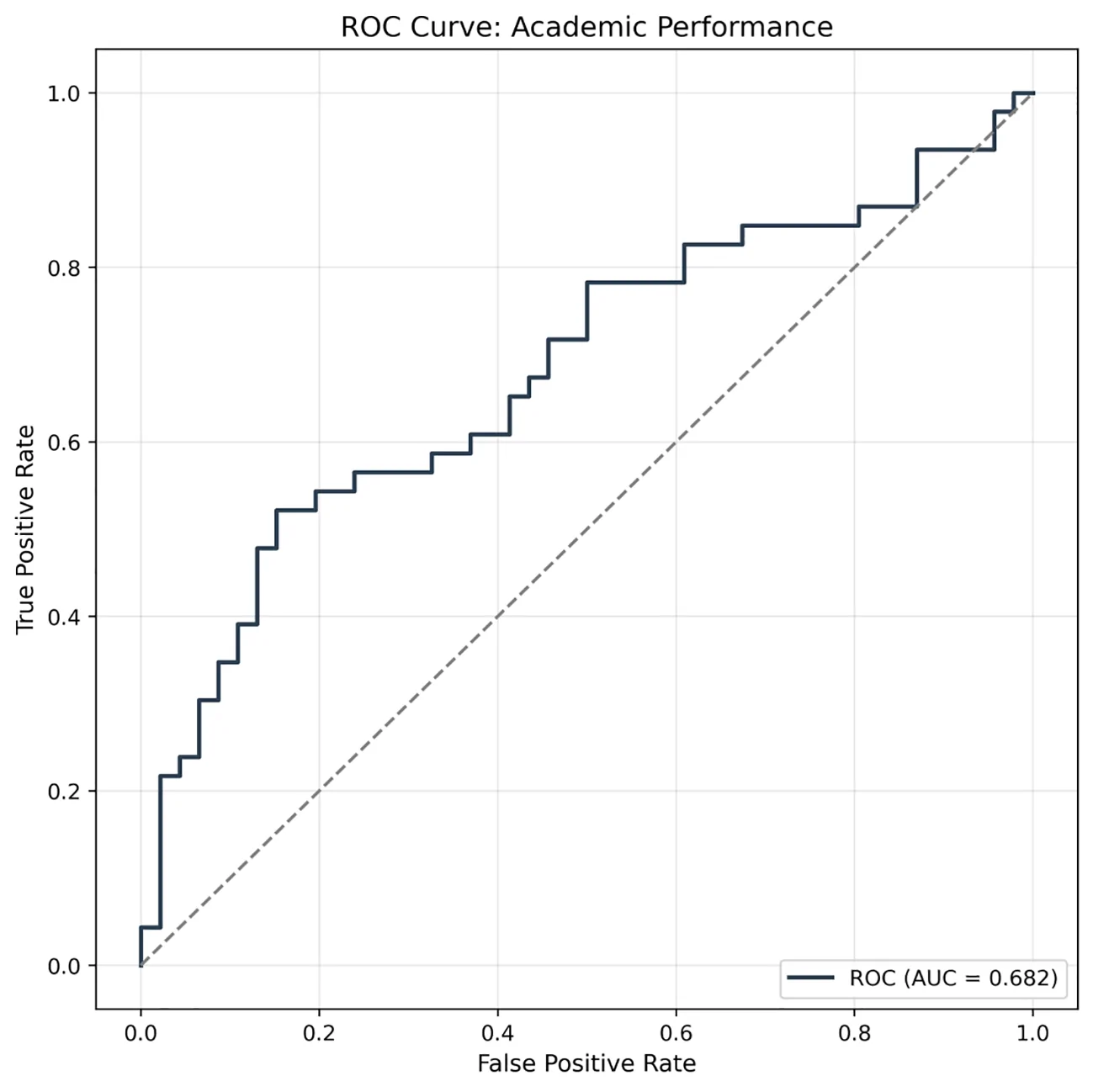
Receiver operating characteristic curve for the academic performance nomogram (cross-validated AUC = .682).

In the academic performance nomogram (**Figures 16**–**19**), gender was the strongest predictor (OR = 2.77), followed by conscientiousness (OR = 1.78) and depression at T0 (OR = 0.60, indicating a protective effect of lower depression). The OCB nomogram identified depression at T0 as the strongest predictor (OR = 0.58), meaning that each standard deviation increase in baseline depression was associated with a 42% reduction in the odds of high OCB. Neuroticism (OR = 0.68) and openness (OR = 1.44) also contributed. The athletic nomogram showed weaker overall prediction, with extraversion as the leading predictor (OR = 1.39).

## 4 Discussion

This study employed four complementary analytical approaches to investigate psychological adaptation in military cadets, progressing from measurement validation through network modelling and person-centred profiling to clinical tool development. The findings offer both theoretical insights into the structure of adaptation processes and practical applications for cadet screening.

### 4.1 Measurement stability

The excellent measurement invariance of the CD-RISC-25 (mean congruence = .971) confirms that resilience, as assessed by this instrument, maintains a stable factorial structure across the training period. This is a critical prerequisite for interpreting longitudinal changes in resilience scores as genuine within-person change rather than measurement artefact, and replicates the good-to-excellent longitudinal psychometric properties of the CD-RISC reported in clinical, community, and military samples (2, 34). The fair invariance of the DASS-21 (mean congruence = .867) warrants more cautious interpretation. The lower T0 versus T1 congruence (.790) suggests that the acute stress of basic training may alter how cadets experience and report distress symptoms, particularly the relative contributions of individual items to their respective subscales. Such item-level shifts under acute environmental stress are consistent with recent psychometric work showing that the DASS-21 factor structure can be sensitive to context and sample composition (12, 13). By T2, the factor structure had largely stabilised, as evidenced by the higher T1 versus T2 congruence (.924), supporting the use of T2 distress scores as substantively comparable to T0 baseline values.

### 4.2 Network structure and bridge nodes

The network analysis revealed that depression and stress formed the strongest and most stable edge in both the cross-sectional and temporal networks. This coupling was present in 100% of bootstrap samples and appeared consistently at all three timepoints, suggesting that interventions targeting either depression or stress would likely affect the other. This finding converges with the broader psychopathology-network literature in which depressive and stress-related symptoms repeatedly emerge as densely interconnected (23, 24), and with the comorbidity evidence underlying the original tripartite formulation of the DASS (11, 33). From a clinical perspective, this implies that stress management programmes during military training may have secondary benefits for depressive symptoms, in line with intervention work showing transdiagnostic effects of stress-regulation training in young adults (49).

The finding that resilience at T0 had the highest betweenness centrality in the cross-sectional network is particularly noteworthy. Betweenness centrality identifies nodes that connect otherwise separate clusters (42), and resilience’s position as a bridge between the distress domain and the personality and organisational behaviour domains suggests that it may serve as a channel through which personality traits influence performance outcomes. This interpretation aligns with theoretical models of resilience as a dynamic process linking dispositional risk factors to adaptive outcomes (15) and with empirical evidence in cadet samples showing that resilience mediates the link between personality and performance (9, 16). The analogous finding for resilience at T1 in the temporal network, which showed the highest betweenness centrality across the three measurement occasions, further indicates that resilience operates as a temporal hinge connecting the early and late phases of training adaptation.

It is worth noting, however, that the network estimates in the present study were derived from a sample of 92 cadets. The 500-iteration non-parametric bootstrap procedure confirmed the stability of the most prominent edges, particularly the depression–stress coupling and the bridging position of resilience, indicating that the principal structural features of the cadet psychological system are robust to resampling. Extension of the analysis to larger longitudinal cadet cohorts would enable finer-grained edge-weight and centrality comparisons, and would allow the observed structure, including the temporal bridging role of resilience, to be examined across a wider range of training contexts.

### 4.3 Cadet profiles

The identification of five distinct latent profiles demonstrates that the cadet population is psychologically heterogeneous, a finding with important implications for training programme design. Rather than assuming a uniform population, training staff may benefit from recognising that approximately half of cadets (Profile 2, 50%) represent a well-adjusted majority, while smaller subgroups show either elevated vulnerability (Profile 4, 10.9%) or distinctive psychological patterns requiring individual attention (Profiles 3 and 5, comprising 10.8% combined). Person-centred designs have repeatedly recovered comparable mixtures of well-adjusted, vulnerable, and atypical profiles in occupational and educational samples (22), and the small but distinct extreme-score profiles identified here mirror subgroups observed in young-adult and military cohorts characterised by high distress and low resilience that are at particular risk for attrition and impaired functioning (1, 2).

The predictive validity of the profile solution was confirmed by significant differences on 17 outcome variables, including OCB, athletic performance, and T2 resilience, none of which were used in profile identification. The large effect sizes for distress variables (depression eta-squared = .605, stress = .497) indicate that profile membership captures substantial variance in psychological outcomes. Notably, the profiles also differentiated on organisational outcomes (OCB eta-squared = .144), suggesting that baseline psychological characteristics have implications for military-relevant behaviour beyond individual wellbeing. This pattern echoes broader evidence that conscientiousness, emotional stability, and resilience predict both in-role performance and discretionary OCB (7, 20, 50).

The change-score sensitivity analysis reframes the role of the primary typology. The two solutions converged on a five-profile structure but shared only modest classification agreement (adjusted Rand index = .245), and the largest effect sizes in the sensitivity solution emerged for the change-score inputs themselves and for concurrent T2 distress, while academic, athletic, and OCB outcomes did not survive Holm correction. Substantively, this pattern suggests that the baseline T0 typology and the trajectory typology characterise distinct facets of cadet heterogeneity: the T0 solution captures who a cadet is at intake and maps onto downstream performance outcomes, whereas the change-score solution captures how a cadet adapts across the training year and is most strongly linked to subsequent distress states rather than to performance. The complementarity of cross-sectional and trajectory-based classifications has been emphasised in recent person-centred reviews (22), and the present results extend it to the military cadet context. The two analyses are therefore complementary, and the primary T0 solution is recommended as the focal typology for screening purposes because of its direct predictive link to operational outcomes.

As is common in person-centred analyses of psychological data, the interpretive scope of the identified typology reflects the size and composition of the sample from which it was derived. The primary solution and its change-score sensitivity counterpart each contained two to three smaller profiles whose mean and effect-size estimates should be regarded as descriptive of the present cohort. The convergent recovery of a five-profile structure under both analytical formulations, together with meaningful differentiation on multiple outcome variables not used in profile identification, nonetheless supports the substantive interpretation offered here. Replication in larger and independent cadet samples would further clarify which components of the profile structure are stable features of first-year cadet populations.

### 4.4 Clinical screening utility

The nomogram results indicate that baseline psychological measures have moderate predictive value for academic performance (AUC = .682) but limited utility for predicting OCB (.544) and athletic performance (.424) using simple logistic regression. The academic model’s superior performance likely reflects the strong association between conscientiousness, gender, and academic achievement, which are well-established predictors in educational psychology (7, 8). The weaker prediction of OCB and athletic outcomes is consistent with findings from the classification pipeline, where more complex machine learning models (Random Forest, SVM) were required to achieve acceptable discrimination, suggesting that these outcomes depend on nonlinear variable interactions that logistic regression cannot capture. This pattern aligns with the broader literature showing that athletic performance is multidetermined and only weakly explained by single psychological predictors (50, 51), and that organisational citizenship behaviour reflects an interaction between dispositional and contextual factors (18, 20).

Despite the modest predictive accuracy of some nomograms, the point-based scoring system offers practical value as a rapid screening heuristic. A cadet scoring high on depression, high on neuroticism, and low on conscientiousness would accumulate negative points across multiple predictors, flagging them for closer monitoring. The nomogram format is intentionally simple, designed for use by training officers without statistical expertise, following the translational philosophy of Iasonos et al. (2008) (25) and the prognostic-model reporting recommendations of Steyerberg and Vergouwe (2014) (31). Routine intake screening of this kind has been recommended in recent military mental-health work as a low-cost mechanism for early identification of at-risk personnel (1, 52).

The predictive accuracy reported for the three nomograms should be interpreted in the light of the training-sample size available in the present study. The models were fitted and internally cross-validated on 92 cadets, and while the resulting discriminative-performance estimates are informative and consistent with the wider literature on personality-based prediction of academic and organisational outcomes, external validation in independent cohorts would provide the strongest basis for operational adoption. The point-based scoring format is deliberately compatible with such validation: it can be applied directly to new samples without further model retraining, and future work applying the nomograms to additional cadet cohorts, whether across successive intakes at the same institution or across other military academies, would consolidate the evidence base for their institutional deployment.

### 4.5 Practical implementation in military training programmes

The findings of the four analytical steps outlined above may translate directly into a coherent framework for the organisation of psychological assessment and support within military academies. The framework is layered rather than uniform, mirroring the structure of the analytical pipeline: intake screening supplies the initial triage, profile assignment refines it, periodic re-assessment tracks its evolution across the training year, and institutional decision-support translates the analytical outputs into forms usable by frontline staff. Each layer is designed to accommodate the practical realities of the training environment, particularly the ceiling on clinician and instructor time and the necessity of preserving cadet trust in the confidentiality of screening data.

At the point of academy entry, the risk nomograms developed in Section 3.4 can serve as a rapid, low-burden triage tool. The BFI-44, DASS-21, and CD-RISC-25 already form part of the standard psychological assessment battery administered at HAFA and comparable institutions, and their operational use as screening instruments requires no additional data collection beyond the point-based scoring of the three composite risk indices for academic, athletic, and organisational-citizenship performance. Cadets whose predicted probability of belonging to the low-performance group exceeds a pre-specified threshold, for example, the lower quartile of the empirical distribution, can then be flagged for closer follow-up during the acute basic-training phase. The empirical basis for this targeting rests on parallel work on the same cohort, in which baseline personality, distress, and resilience jointly predicted academic, athletic, and composite performance under a multi-model explainable machine-learning framework, with resilience emerging as the most consistent domain-general predictor across all three outcomes (37). Importantly, the same work demonstrated that a gender-neutral composite classification model, in which gender was excluded from the predictor set by design, achieved a cross-validated ROC-AUC of 0.768 for holistic cadet performance (37), indicating that operationally fair prediction of overall training success is achievable without recourse to gender as a screening variable.

For cadets identified at intake as being at elevated risk, application of the latent-profile classifier developed in Section 3.3 refines the triage decision by locating each cadet within the five-profile typology. This step distinguishes cadets whose vulnerability reflects a coherent adverse psychological configuration (Profile 4, the elevated-distress subgroup) from those with distinctive but atypical patterns requiring individualised assessment (Profiles 3 and 5), and from those whose baseline scores approach the risk threshold from within a broadly well-adjusted profile. Each profile suggests a substantively different mode of support. For Profile 4 cadets, peer-mentoring and structured stress-regulation programmes are indicated, and randomised evidence in officer cadets has demonstrated that group-format resilience training combining coping and emotion-regulatory self-reflection can produce measurable gains in resilience over the course of a single training cycle (17). For Profiles 3 and 5, individual case-by-case formulation and psychological intervention are more appropriate, given the atypical configuration these profiles present. For the well-adjusted majority, general wellbeing monitoring is sufficient. This layered use of the typology also aligns with the trait-specific pattern of resilience mediation identified in parallel work on the same cohort, in which resilience was found to mediate the association between agreeableness or leadership on the one hand and psychological distress on the other, whereas neuroticism and conscientiousness operated through direct, non-mediated pathways (35). Substantively, this implies that resilience-focused interventions are most likely to benefit cadets in whom resilience already serves an active mediating role, whereas cadets with high neuroticism will additionally require interventions targeting emotion regulation, cognitive reappraisal, and help-seeking stigma.

The network analysis reported in Section 3.2 identified resilience as a temporal hinge connecting the early and late phases of training adaptation, and the measurement-invariance analysis in Section 3.1 confirmed that CD-RISC-25 scores are metrically comparable across the three assessment occasions. Together, these findings support the routine re-administration of the CD-RISC-25 and DASS-21 at the end of basic training (T1) and at the end of the first semester (T2), with staff attention directed to cadets whose resilience trajectory declines or whose distress trajectory rises between waves. Parallel longitudinal work on the same cohort has identified basic training itself as the primary period of psychological change, with significant group-level reductions in distress and increases in resilience observed between T0 and T1, alongside considerable individual variability in trajectory shape (36). The same work further demonstrated, using random-intercept cross-lagged panel modelling, that higher distress predicts lower subsequent resilience (β = −.223 to −.286), whereas the reverse temporal pathway did not emerge (36). Trajectory-based monitoring of the kind envisaged here is therefore not only more informative than any single-timepoint assessment but is also directly targeted at the specific mechanism through which resilience resources are eroded, in a manner consistent with the continuous-monitoring rationale advanced in recent military basic-training research (4).

The point-based nomogram format is deliberately simple, requiring neither statistical software nor specialist training to apply. In operational deployment, a printed nomogram card or a short spreadsheet template would enable resident advisors, clinicians, or training officers to compute a cadet’s predicted-risk score during a routine intake interview and to record the resulting triage decision in the academy’s existing case-management system, in line with the translational philosophy that has underpinned the widespread adoption of prognostic nomograms in clinical medicine (25) and with the prognostic-model reporting standards articulated in the wider prognostic-modelling literature (31). The profile classifier can similarly be embedded within an existing electronic screening platform, either as a downloadable spreadsheet returning the nearest profile or, at higher levels of institutional infrastructure maturity, as a small web-based tool. The gender-neutral composite classification model developed in parallel work on the same cohort (37) provides an additional, complementary decision aid for cases in which a holistic, rather than domain-specific, risk assessment is required.

The parallel mediation–moderation analysis on the same cohort further revealed that leadership operates as a significant protective factor against psychological distress in male cadets but shows a directionally reversed pattern in female cadets, a crossover interaction consistent with role-incongruence dynamics in a predominantly male institutional environment (35). This finding, together with the substantial female–male distress differential (Hedges’ g = −0.60 to −1.10) documented in the same work in the complete absence of corresponding gender differences in personality, resilience, or leadership (35), warrants explicitly gender-informed elements within any operational deployment of the present framework. Leadership-development programmes should be designed and evaluated separately by gender rather than adopting a uniform curriculum, and institutional responses to the acute distress observed among female cadets during basic training should extend beyond dispositional screening to include gender-sensitive onboarding, visible female role modelling, and structural destigmatisation of distress reporting — an approach consistent with the broader literature on secure-base leadership as a moderator of training strain (21).

Any implementation of psychological screening in a military training environment must balance early identification of at-risk cadets against the risks of labelling, stigma, and inappropriate use of screening information in career decisions. The framework outlined here is intended to inform psychological and educational support, not selection, evaluation, or promotion decisions, and its operational deployment would require corresponding safeguards: separation of screening records from academic and disciplinary files, clear consent procedures for the use and retention of psychological information, and periodic audit of the screening decisions against actual training outcomes. Institutional adoption would ideally be accompanied by staff training on the responsible interpretation of psychological screening data and by ongoing dialogue with cadets on how their psychological information is used, following the reporting and transparency standards articulated for prognostic models in clinical settings (31).

The framework has been deliberately designed to minimise its footprint within existing training routines. All measurement waves involve well-validated self-report instruments that can be administered in group format within a single 30-minute session, all analytical outputs can be scored and interpreted by academy mental health staff without further computational infrastructure, and the entire framework maps onto milestones already present in the academic and physical training calendar (entry, end of basic training, end of first semester). This design choice reflects the practical realities of military training, where any additional burden on cadets or staff must be justified by clear operational benefit, and is consistent with recent recommendations to embed low-burden psychological monitoring into the routine architecture of military basic training rather than as a separate parallel process (1, 4).

## 5 Conclusions, limitations, and future directions

### 5.1 Conclusions

The present study set out to map psychological heterogeneity in a cohort of military academy cadets by integrating four complementary analytical perspectives within a single longitudinal dataset. Across the four analyses, a coherent picture emerged. Measurement invariance testing confirmed that the CD-RISC-25 provides stable assessments of resilience across the training period, supporting its use as a longitudinal monitoring instrument, whereas the DASS-21 showed fair but imperfect stability that requires cautious interpretation of T0-versus-T1 comparisons during basic training. Network analysis revealed that depression and stress form the densest and most stable connection in cadet psychopathology, while resilience occupies a bridging position that connects the distress domain to the personality and organisational citizenship domains, and at T1 connects the early and late phases of training in the temporal network. Latent profile analysis identified five distinct cadet types, ranging from a well-adjusted majority to small vulnerable and atypical subgroups, and showed meaningful differentiation on 17 outcome variables not used in profile construction. A pre-specified sensitivity analysis using change-score inputs reproduced a five-profile structure but yielded only modest concordance with the primary T0 solution (adjusted Rand index = .245), supporting the interpretation that baseline configuration and longitudinal trajectory capture complementary, non-redundant facets of cadet heterogeneity. Finally, three logistic regression nomograms provided a deployable screening framework, with the academic model achieving the highest cross-validated discrimination.

Taken together, these findings advance the study of cadet adjustment in three concrete ways. First, they confirm that a small set of psychologically meaningful indicators, particularly conscientiousness, neuroticism, baseline distress, and resilience, are jointly informative about training outcomes across academic, athletic, and organisational domains, in line with both meta-analytic personality-performance evidence (7, 50) and cadet-specific work (9, 16). Second, they show that the cadet population is not psychologically uniform: distinct subgroups with qualitatively different configurations exist, and these subgroups have different prognoses for both wellbeing and performance (22). Third, they demonstrate that the gap between predictive modelling and clinical deployment can be bridged with minimal loss by translating empirical findings into nomograms that are interpretable, additive, and usable by frontline staff without statistical training (25, 31).

From a practical standpoint, the results suggest a layered approach to cadet support. Routine intake screening based on the nomograms can flag cadets at elevated risk for poor academic outcomes. Profile assignment can then refine this triage by distinguishing between vulnerable, atypical, and well-adjusted subgroups, each potentially benefiting from a different support strategy. Periodic re-measurement of distress and resilience across T0, T1, and T2, supported by the network-analytic evidence that resilience is a temporal hinge, allows training staff to detect deteriorating trajectories before they manifest in performance failure or attrition (1, 4). The change-score sensitivity LPA further indicates that trajectory typologies, although not directly tied to performance, may be valuable for distress-focused monitoring. Beyond the practical implications, the multi-method framework illustrated here can be exported to related high-demand training environments, including officer-candidate schools, elite sport academies, and emergency-services training programmes, where the same combination of individual differences, acute distress, and resilience trajectories is likely to be operative.

### 5.2 Limitations

Several limitations should be considered when interpreting these findings. First, the sample size of 92 cadets, while sufficient for the analyses conducted, limits statistical power and the generalisability of results. The five-profile LPA solution included two profiles with fewer than six members, and these small subgroups should be interpreted with caution pending replication in larger samples. The implications of this sample-size constraint differ modestly across the four analyses. In the network analysis, the strongest edges and centrality patterns were confirmed by bootstrapping, but finer-grained comparisons would benefit from larger samples. In the latent profile analysis, the smaller profiles are more sensitive to sample composition, and their characterisation should be regarded as descriptive of the present cohort. In the risk nomograms, external validation in independent cohorts would strengthen the operational recommendations. Replication across additional institutions and successive intake cohorts would consolidate the generalisability of these findings, while the present study, through its integration of measurement-invariance validation, bootstrapped network stability, sensitivity-tested profile identification, and internally cross-validated screening models, establishes an internally rigorous baseline against which such replication can proceed. Second, the study was conducted within a single cohort at one military academy, and the profile structure and network topology may differ in other training contexts or cultural settings. Third, the DASS-21 showed lower measurement invariance during basic training, suggesting that comparisons of absolute distress levels between T0 and T1 should be made cautiously. Fourth, the nomograms used only baseline (T0) predictors, and their moderate predictive accuracy reflects the inherent difficulty of prospective prediction using pre-training measures alone. Fifth, the graphical LASSO network estimation assumes multivariate normality and may not fully capture nonlinear relationships among variables.

### 5.3 Future directions

Future research should aim to replicate the five-profile solution in independent and larger cadet cohorts to assess its generalisability. The integration of ecological momentary assessment data could provide more granular temporal resolution for network analysis, capturing within-day fluctuations in distress and resilience. The development of dynamic nomograms that incorporate T1 measures alongside T0 predictors may substantially improve prediction of T2 outcomes. Additionally, intervention studies could test whether targeting resilience, the identified bridge node, produces the cascading effects across the distress and performance domains that the network structure suggests. Finally, the application of Bayesian structural equation models could address the small sample limitations and provide more robust parameter estimates with credible intervals.

## Data Availability

The datasets presented in this article are not readily available due to the institutional confidentiality requirements of the Hellenic Air Force Academy. Requests to access the data can be directed to the corresponding author for consideration by the relevant authority.
